# Splitting the leafmining shield-bearer moth genus *Antispila* Hübner (Lepidoptera, Heliozelidae): North American species with reduced venation placed in *Aspilanta* new genus, with a review of heliozelid morphology

**DOI:** 10.3897/zookeys.957.53908

**Published:** 2020-08-10

**Authors:** Erik J. van Nieukerken, Charles S. Eiseman

**Affiliations:** 1 Naturalis Biodiversity Center, PO Box 9557, NL-2300 RA Leiden, The Netherlands Naturalis Biodiversity Center Leiden Netherlands; 2 276 Old Wendell Rd., Northfield, MA 01360, USA Unaffiliated Northfield United States of America

**Keywords:** Canada, DNA barcodes, grapevine pest, *
Heliozela
*, phylogeny, United States, Vitaceae

## Abstract

The new genus *Aspilanta***gen. n.** is described to harbour Nearctic heliozelid moths with reduced venation, previously placed in *Antispila* Hübner, 1825, with type species *Antispila oinophylla* van Nieukerken & Wagner, 2012. The erection of this genus has become possible now that monophyly has been supported by a recent phylotranscriptomics analysis. Six species are combined in this genus: *Aspilanta oinophylla* (van Nieukerken & Wagner, 2012), **comb. n.**, *A. hydrangaeella* (Chambers, 1874), **comb. n.**, *A. ampelopsifoliella* (Chambers, 1874), **comb. n.**, *A. voraginella* (Braun, 1927), **comb. n.**, *A. argentifera* (Braun, 1927), **comb. n.**, *A. viticordifoliella* (Clemens, 1860), **comb. n.** and two candidate species are recognised. DNA barcode COI sequences of Malaise trapped specimens suggest a rich fauna of *Aspilanta* in Central America. All are leafminers, with Vitaceae as main host family, and single species feeding respectively on Hydrangeaceae and Myricaceae. The species are briefly diagnosed, and data on biology, DNA barcodes and distribution are provided. To place the genus in context, a review of heliozelid morphology and phylogeny is presented and a key to Nearctic genera is given. The genus is confined to North and Central America, possibly also occurring in South America. *Aspilanta oinophylla* is also an invasive species on grapevine in Italy. The genus is sister to *Coptodisca* Walsingham, 1895. Another species is removed from *Antispila*: *Heliozela eugeniella* (Busck, 1900), **comb. n.**, feeding on *Eugenia* (Myrtaceae), from Florida.

## Introduction

The taxonomy of the Lepidoptera family Heliozelidae, the shield bearer moths, has recently received considerable attention due to the discovery of several previously unknown pest species, attacking grapevines and walnuts (van Nieukerken et al. 2012; Bernardo et al. 2015; van Nieukerken and Geertsema 2015; Wang et al. 2018; Takács et al. 2020), as well as the discovery of a rich unknown fauna in Australia that includes pollinating species (Milla et al. 2017). Through international collaboration, it became possible to study the phylogeny of the family on the basis of a large selection of taxa with some nuclear and mitochondrial genes (Milla et al. 2017), and for a smaller subset with transcriptomes (Milla et al. 2019).

It soon became apparent that the relatively large genus *Antispila* Hübner, 1825, was a catch-all genus for species similarly patterned with a fascia before the middle of the forewing and a pair of opposing spots (the meaning of the Greek anti-spila / ἀντί-σπίλα) in the distal half. Several African and Asian species in *Antispila* with a reduced venation were transferred to the previously monotypic genus *Holocacista* Walsingham & Durrant, 1909 (van Nieukerken and Geertsema 2015). The Nearctic species that invaded Italy, *Antispila oinophylla* van Nieukerken & Wagner, 2012 and its relatives, share a similar reduced venation, but without a phylogenetic generic framework it was still described in *Antispila*, although in the original description it was made clear that this was a temporary placement (van Nieukerken et al. 2012). The phylogeny in Milla et al. (2017) showed that all taxa with reduced venation form a clade: the genera *Holocacista, Antispilina* Hering, 1941, *Coptodisca* Walsingham, 1895 together with several species in *Antispila*, whereas the type species of *Antispila*, *A. metallella* ([Denis & Schiffermüller], 1775) and other species with the full venation form another clade. However, in that study, the species with reduced venation in *Antispila* were paraphyletic with respect to *Coptodisca* and formed two clades: the *A. ampelopsifoliella* group and *Antispila group* II. The transcriptomics study, in contrast, showed a strong support for monophyly of the sampled representatives of these groups, here *A. oinophylla* and *A. argentifera* Braun, 1927. As the continued use of the incorrect generic name *Antispila* can cause confusion, we describe here a new genus for this group of species and give the invasive grapevine miner *A. oinophylla* its correct taxonomic placement.

To place this description in a wider context, we review the taxonomic history of the genus *Antispila* sensu lato and review knowledge on the morphology and phylogeny of the family Heliozelidae, partly based on the unpublished thesis of the late Ebbe Nielsen (1980).

### Taxonomic history of *Antispila* Hübner

In an attempt to divide the large genus *Tinea* into smaller entities, at the time used for almost all small microlepidoptera, Hübner (1816–1826 [1825]: 419) described the genus *Antispila* for a group of species with opposite forewing spots (“Die Schwingen mit entgegengesetzten hellen Flecken oder Bändchen angelegt”). He included 13 species, of which only his *A. Stadtmüllerella* (given as replacement name for his *Tinea Pfeifferella*) is a heliozelid. Other included species belong now to Micropterigidae (3), Eriocraniidae (1), Nepticulidae (1), Meessiidae (1), Elachistidae (3), Oecophoridae (1), and the placement of two is unknown. Hübner’s genus was not immediately recognised by other European authors, who usually placed species that are now in *Antispila* in other genera, such as *Elachista* Treitschke, 1833 (Fischer von Röslerstamm 1834–1843; Stainton 1851,^1854^) or *Oecophora* Latreille, 1796 (Duponchel 1843).

The first to restrict the genus to heliozelid species was Herrich-Schäffer (1855), who recognised two species: *A. treitschkiella* (Fischer von Röslerstamm, 1843) and *A. pfeifferella* (Hübner, 1813), implicitly using *A. Stadtmüllerella* (as *pfeifferella*, a junior synonym of *A. metallella* (Denis & Schiffermüller, 1775)) as type species, although no formal selection was made according to the current rules (ICZN 1999). He also introduced the name *Heliozela* Herrich-Schäffer, 1853, and placed the two genera together (Herrich-Schäffer 1853). This view on *Antispila* was then followed widely in Europe in the 19^th^ and 20^th^ centuries (Frey 1856; Stainton et al. 1870; Meyrick 1895; Spuler and Meess 1910; Heinemann and Wocke 1876), although Frey still also included what we now know as *Stephensia brunnichella* (Linnaeus, 1767) (Elachistidae). Heinemann and Wocke (1876) also recognised and named the family Heliozelidae for the first time.

In North America, Clemens discovered *Antispila* leafmines on “*Nyssa multiflora*” (black gum, now known as *Nyssa sylvatica*, Nyssaceae), but at first did not know where the reared moths belonged and suggested in a letter to Stainton on 15 May 1859 the new name *Diacopia*. This name has unintentionally, but validly been published as *Diacopia* Clemens, 1872, when Stainton published their correspondence much later (Clemens and Stainton 1872). Undoubtedly Stainton informed Clemens about the correct position of this species, and shortly thereafter the first four Nearctic species were described in *Antispila* by Clemens (1860a, 1860b); later more by Chambers (1874a, 1874b), Busck (1900), Braun (1915,1927a, 1927b) and Lafontaine (1973).

A formal selection of *Antispila stadtmuellerella* as the type species was made by Fletcher (1929), but unfortunately, he and most later authors overlooked the fact that Hampson (1918) had already made a selection of the first species in Hübner’s list: *A. pagenstecherella* (Hübner, 1825). However, this species was already recognised in the genus *Eudarcia* Clemens, 1860, then Tineidae, now in Meessiidae. Because of this unfortunate situation, where the well-known generic name *Antispila* in fact belonged to another family, Nielsen and Nye (1986) applied to the International Commission on Zoological Nomenclature to validate Fletcher’s selection of the type species rather than Hampson’s. This was granted two years later (ICZN 1988).

Until recently, the validity of the genus *Antispila* was rarely challenged; only the small Mediterranean species *A. rivillei* (Stainton, 1855) was moved to the genus *Holocacista*, when the different venation was noted by Walsingham (1909). Nielsen and Traugott-Olsen (1981) moved the American *A. major* Kearfott, 1907 to *Stephensia* (Elachistidae) and later one more misplaced Elachistidae was discovered: *A. merinaella* Paulian & Viette, 1955 from Madagascar, without assigning it to another genus (van Nieukerken and Geertsema 2015).

The presence of species with a reduced venation was already noted by Meyrick (1916) when describing the Neotropical *A. trypherantis* Meyrick, 1916, *A. pentalitha* Meyrick, 1916 and later *A. cyclosema* Meyrick, 1921 (Meyrick 1921). This was also noted by Kuroko (1961), when describing the first Japanese *Antispila*. In North America, Don Lafontaine (pers. comm.) had also independently discovered the difference in venation, and a difference in the shape of the “shield”, the pupal case cut out from the leaf mine by the mature larva, when working on Heliozelidae, and planned the description of a new genus in the early 1970s; however, this never happened as he changed his study subject to larger moths.

When describing the new *Antispila oinophylla*¸ the non-monophyly of North American *Antispila* became apparent once again (van Nieukerken et al. 2012), and during the study of South African Heliozelidae, several African and Indian species were transferred from *Antispila* to *Holocacista* and *Heliozela* respectively (van Nieukerken and Geertsema 2015). The two phylogenetic studies that followed (Milla et al. 2017, 2019) corroborated this and strongly indicated the polyphyly of the old genus *Antispila*. Here we continue this process by placing six *Antispila* species in the new genus *Aspilanta* and recombining one species with *Heliozela*. After this action, five named Nearctic species remain in *Antispila* s. str., three feeding on *Cornus* (Cornaceae) and one each on *Nyssa* (Nyssaceae) and *Vitis* (Vitaceae). Globally the genus now has 27 named species in the Holarctic and Oriental regions, mainly feeding on Cornales and Vitaceae. The three European species, all feeding on *Cornus*, were recently revised by van Nieukerken et al. (2018); the 12 named and one unnamed Japanese species were treated by Lee and Hirowatari (2013) and in total five species were recently described from China (Liu and Wang 2017; Wang et al. 2018; Liao et al. 2019). One unnamed species was recorded from South Africa (van Nieukerken and Geertsema 2015). The majority of the nine species listed in *Antispila* from the Neotropics (Heppner 1984) probably belong in other genera, including *Aspilanta*.

## Materials and methods

As most species treated here were already discussed in the paper describing *Antispila oinophylla*, we re-use several photographs from van Nieukerken et al. (2012) and refer to that paper for the methodology used by EvN for collecting, preparation, imaging, and DNA barcoding, and updates in subsequent papers on Heliozelidae (van Nieukerken and Geertsema 2015; van Nieukerken et al. 2018). Additional fieldwork in North America was carried out by EvN in 2015, 2016 and 2018.

All examined and observed material, including parasitoids and all available barcoding data are listed in the occurrence dataset, published with GBIF (DOI: https://www.doi.org/10.15468/79mu7z). In the text we omit all material that was already listed by van Nieukerken et al. (2012), even though some of these records later received registry numbers (in CNC and RMNH) or were dissected; these data can be found in the GBIF dataset. Material collected in 2011 was cited in the 2012 paper only from larvae and leafmines; adults reared in 2012 are cited here. We follow the new guidelines for fine-grained formatting of material examined. We divide the material into four categories: examined adults (numbers without male or female sign represent unsexed adults); examined larvae and leafmines; DNA barcoded material that we did not examine personally, but identified on the basis of the barcode (all DNA barcoded specimens can also be found in the BOLD dataset DS-ASPIL (DOI: https://www.doi.org/10.5883/DS-ASPIL); and observations from internet sites and other photographs that could be reliably identified on the basis of the photograph; unless otherwise indicated, these refer to leafmines with or without larvae.

Under the heading “Distribution” we list countries, states and provinces; an asterisk denotes a new record.

Leafmines were photographed by CSE in the field using a Nikon D50 digital camera and a Nikon AF Micro Nikkor 105 mm lens. The camera’s built-in flash was used for photographs with reflected light; the sun was used for transmitted light. All other photographs by CSE were taken using a Canon EOS Rebel XSi SLR digital camera, MP–E 65 mm macro lens, and Macro Twin Lite MT–24EX flash unit. For transmitted light in leaf mine photographs, one of the flash heads was removed and aimed at the camera while the leaf was held tightly against it.

Leaves containing larvae were placed in sealed, 9– or 15–dram plastic vials, which were stored away from direct sunlight and checked daily (when possible) for emerging insects. After pupal cases were formed, extraneous leaf material was removed from the vial to minimise mould and a moistened, crumpled piece of toilet paper was added to prevent desiccation. Overwintering pupal cases were stored in a refrigerator at 1–3 °C from 6 November 2013 to 25 February 2014, 1 November 2014 to 1 March 2015, 1 November 2015 to 1 March 2016, 20 October 2017 to 1 April 2018, and 17 October 2019 to 21 February 2020. Adult moths were chilled and photographed as described by Eiseman and Lonsdale (2018) for agromyzid flies.

Morphological terminology follows van Nieukerken et al. (2012) with the corrections proposed by van Nieukerken and Geertsema (2015).

Host plant names follow the Catalogue of Life (Hassler 2020), which agrees with the Flora of North America for the Vitaceae (Moore and Wen 2016) and Hydrangeaceae (Freeman 2016), but for Myricaceae rather follows Wilbur (1994). As the differences between the Virginia creeper species *Parthenocissus quinquefolia* and *P. vitacea* are rather subtle, it is possible that some records of *P. quinquefolia* may in fact refer to *P. vitacea*. According to Pringle (2010) the last species should be named *P. inserta*, but the cited sources do not follow this, despite the rather strong argumentation.

We list parasitoids that we reared from *Aspilanta* larvae. Those reared by EvN were routinely stored in ethanol 96–100%, and later DNA barcoding was performed (dataset DS-PARASP, doi: https://www.doi.org/10.5883/DS-PARASP). Chalcidoidea were morphologically identified by Sandrine Ulenberg, Braconidae were tentatively identified by EvN on the basis of DNA barcodes; all are stored in RMNH; Barcode Identification Numbers are listed after the taxon names. Parasitoids reared by CSE were photographed alive, then preserved in 95% ethanol and sent to specialists: all Eulophidae, except the *Pediobius*, were examined by Christer Hansson and deposited in BMNH; Braconidae were determined by José Fernández-Triana from photographs and are deposited at CNC. We have not found any literature records of reared parasitoids that were identified below family level. Species listed here as Braconidae: Microgastrinae belong either to the genus *Dolichogenidea* or *Pholetesor*; a detailed taxonomic revision of this group is necessary (José Fernández-Triana, pers. comm.). The record of this subfamily listed here under *A. ampelopsifoliella*, was in fact the basis for the first record of Heliozelidae as host family (Fernandez-Triana et al. 2020). All records of parasitoids are also listed in the GBIF dataset (DOI: https://www.doi.org/10.15468/79mu7z).

Abbreviations for depositories:

**ANSP**Academy of Natural Sciences in Philadelphia, Pennsylvania, USA.

**BIOUG**Centre for Biodiversity Genomics, Guelph, Canada.

**BMNH**Natural History Museum, London, UK.

**CNC**Canadian National Collection of Insects, Arachnids and Nematodes, Ottawa, Canada (registration numbers start with CNCLEP).

**CSEC** Personal collection of C.S. Eiseman.

**MCZ**Museum of Comparative Zoology, Harvard University, Cambridge, Massachusetts, USA.

**NFRC**Northern Forest Research Centre, Forestry Canada, Edmonton, Canada.

**RMNH**Naturalis Biodiversity Center, Leiden, Netherlands (registration numbers start with RMNH.INS).

**TLMF**Tiroler Landesmuseen, Ferdinandeum, Hall, Austria.

**USNM**Smithsonian National Museum of Natural History, Washington DC, USA.

**ZMUC**Zoological Museum, Natural History Museum of Denmark, Copenhagen, Denmark.

## Taxonomy

### Genus Aspilanta gen. n.

Taxon classificationAnimaliaLepidopteraHeliozelidae

DA371C2B-878A-5F83-A346-E02D9EE0BF83

http://zoobank.org/897EE415-F1AF-46A8-9404-49EFC6E2D80A

Antispila : auct. partim, nec Hübner, 1825.
Antispila ampelopsifoliella group; van Nieukerken et al. 2012: 66.
Antispila “group II”; Milla et al. 2019: 133.

#### Type species.

*Antispila oinophylla* van Nieukerken & Wagner, 2012: 38, by present designation.

#### Differential diagnosis.

Very small moths, wingspan between 4.0 and 6.2 mm, with a forewing pattern of metallic–silvery markings, comprising an oblique fascia at ¼, a postmedial pair of spots (one costal and one dorsal), and usually a small apical spot (only absent in *A. viticordifoliella*); fringe line more or less distinct. Males never with androconial scales or hair-pencils. Antennae with only 16–20 segments. *Antispila* species in North America never have an apical spot. *Aspilanta* species are diagnosed by the reduced venation (Figs 9–13); in *Antispila* the discoidal cell is present and more veins are retained (illustrated in van Nieukerken et al. 2012; van Nieukerken et al. 2018); most *Antispila* species are larger and have more antennal segments. Separated from *Heliozela* species by more extensive colour pattern and apical spot, and *Heliozela* species have the venation with discoidal cell and a distinct epiphysis on foreleg. *Coptodisca* species are readily recognised by their colour pattern (Bernardo et al. 2015; Eiseman 2019). The genera *Holocacista* and *Antispilina*, not yet known from the New World, have a very similar venation, but always lack the apical spot. Moreover, *Holocacista* species have a small epiphysis on the foreleg, and the phallus bears an unusually long, often recurved appendix.

#### Description.

**Adults** (Figs 1–8, 45–50). Very small moths, forewing length ca. 1.8–2.8 mm (wingspan ca. 4.0–6.2 mm), no sexual dimorphism.

*Head* (Figs 14–18). Almost oval in outline. Eyes in latero-ventral position, ventral margin not reaching lower margin of head. No sutures present. Anterior tentorial arms very slender, prominently curved laterally before converging towards frons. Vestiture comprising lamellar scales, closely appressed on head, in dry specimens scales on vertex sometimes raised, probably an artefact as a result of drying. Mouthparts: labrum narrow, pilifers absent. Mandibles small, as long as broad, relatively well sclerotised (Fig. 16). Maxilla with galea well developed and almost twice as long as head; maxillary palp reduced to a single segment. Labial palp well developed, 3-segmented, drooping, slightly shorter than head capsule; distal segment almost 3× as long as second segment; depression for Organ von Rath not seen. Antenna (Figs 14, 18) ca. half length of forewing with 14–18 flagellomeres (16–20 segments) [best counted in denuded specimens on slides], no sexual dimorphism. Scape and pedicel of equal length, slightly shorter and wider than flagellomeres. Flagellomeres (Fig. 18) cylindrical, ca. twice as long as wide, each with two annuli of scales, most dark, some apical flagellomeres may be white. Pecten present, but not easily visible; with ca. 4–5 hairs.

*Thorax*. Vestiture of appressed lamellar scales, either concolourous with ground colour of forewings or more metallic and similar to vestiture of head. Foreleg (Fig. 20) without epiphysis.

*Wings*. Male retinaculum a series of 7–12 hook-shaped bristles (Fig. 19), arising from a thickened serrate portion of Sc. Frenulum in male a strong curved bristle (Figs 12, 13), in female two bristles present (Fig. 10); pseudofrenular bristles in male absent. Humeral field with scattered microtrichia, otherwise microtrichia restricted on wing membrane to area just posterior to retinaculum, arranged in longitudinal rows. Scale sockets regularly spaced, not in distinct rows.

*Venation* (Figs 9–13). Forewing with Sc to middle of costa. R unbranched, a separate vein, to costa, but a persistent trachea connecting R with Rs+M+CuA. Rs+M+CuA ending in 3–4 branches, interpreted as Rs1+2 to costa, Rs3+4 to termen, M and CuA to dorsum. Hindwing with Sc+R to costa, Rs+ M with 2–3 branches, Rs to costa, 1 or 2 branches of M to termen and dorsum; CuA a separate vein to dorsum.

**Figures 1–8.**
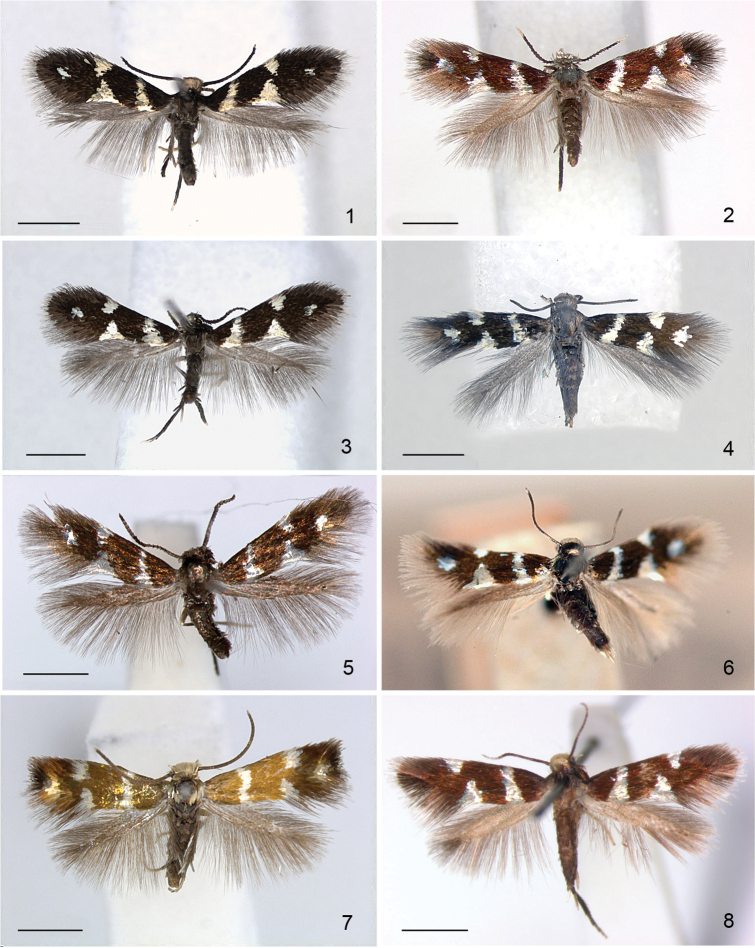
*Aspilanta* species, mounted adult moths. **1***A. oinophylla*, male, USA, VT, RMNH.INS.24378 **2***A. hydrangaeella*, female, USA, NC, RMNH.INS.25191 **3***A. ampelopsifoliella*, male, USA, CT, RMNH.INS.24377 **4***A argentifera*, female, USA, MA, RMNH.INS.25019 **5***A. voraginella*, male, USA, AZ, RMNH.INS.23918 **6***A. argentifera*, female holotype **7** Undescribed species in unnamed genus14 (“Neospila”), male, Chile, Osorno, 20 km W of Entre Lagos, 17.x.1981, leg. Nielsen & Karsholt, genitalia slide EJvN 4632, ZMUC**8***A. viticordifoliella*, female, Canada, Ottawa, from *Parthenocissus*, CNCLEP00122404. Scale bars: 1mm.

*Wing pattern* (Figs 1–8). On forewing typically comprising a silvery white metallic fascia at 1/3, widest at dorsum, and a similarly coloured pair of opposite spots at 2/3 on a dark background, brown to black, with a small silvery blue spot in apex, equidistant to dorsum, costa and fringe line, but the apical spot is absent in *A. viticordifoliella*. A fringe line often present, with fringe scales pale. Hindwing uniform grey. Androconial scales absent in all species examined.

*Pregenital abdomen*. Abdominal sclerites weakly sclerotised. Anterior sternum II subtriangular, free.

*Male genitalia* (Figs 21–28). Vinculum (SIX) very long, anteriorly often reaching beyond anterior margin of segment VI, almost cylindrical; approximately 2/3 of total length of genitalia. Tegumen (TIX) narrow, often bilobed, or medially indented, or truncate; probably a composite structure with uncus. Gnathos absent. Valva approximately triangular, with stalked pectinifer halfway to inner margin, pecten comprising 10–22 blunt sensilla (comb teeth); transtilla typically with medial anterior projection, sublateral processes long. Phallocrypt (manica) with some to many strongly sclerotised conical spines, often arranged in an asymmetric fashion, or with many smaller spines. Phallus outer tube often with remarkable appendices of different sizes and shapes. Juxta present, often bilobed or arrow shaped, in *hydrangaeella* with extra spines laterally.

*Female genitalia* (Figs 29–33). SVIII truncate, TVIII deeply indented. Oviscapt with few lateral cusps. Anterior and posterior apophyses subequal in length, a short interapodemal process between anterior apophyses (Fig. 29). Spermathecal papilla usually with circular sclerotisation. Ductus spermathecae with several coils.

*Larva* (Figs 34–42). Yellow or whitish, usually with brown to almost black head capsule and prothoracic sclerites and dark anal plates; no plates present on other segments, but concentration of cuticular swellings can give impression of darker plates on abdomen in some species. Head prognathous, 2 stemmata at either side. Thorax with elongate dorsal and ventral sclerites without adornment; 10^th^ (last) abdominal segment with single dorsal and paired ventral sclerites, with several prominent setae; other thoracic and abdominal segments covered with small transverse swellings. Legs and prolegs absent but paired ambulatory calli on T2 and T3 (ventral and dorsal) and fused ventro-medial calli on A3–6. Number of instars unknown, but likely with four feeding instars and a fifth non-feeding instar that constructs the case in which it pupates, in analogy to *Holocacista*, *Coptodisca*, *Antispila* and *Heliozela* (Dziurzyński 1948, 1952; Marchi 1956; Prota 1962; Maier 1988).

#### Biology.

Host plants. Most species feed on Vitaceae, one each on Hydrangeaceae and Myricaceae.

#### Life history.

Eggs are inserted in leaf tissue, often near a vein or leaf margin. All species construct leafmines, either starting as a narrow linear mine and later widening into a blotch, or sometimes starting almost immediately as a blotch mine. All frass is deposited in the mine, initially filling or irregularly scattered in the linear portion, later often scattered in the blotch or pushed by the larva to one side. During the penultimate (fourth) instar an oval section is cut out from both epidermal layers, forming a portable case or “shield”. This shield (Figs 55, 68, 76, 93, 106), later forming the cocoon, is more or less flat, without the raised central ridge that is characteristic for *Antispila*. The larva descends with its shield on a strand of silk and may wander for some distance before finally attaching the shield at one end to a substrate (leaf, trunk, leaf litter, etc.), where it moults to the fifth non-feeding instar and later pupates. The pupa protrudes from the opposite end of the shield when the adult emerges. As far as we know, most species are bivoltine, overwintering as fifth instar larva in the cocoon, but *A. voraginella* and possibly *A. ampelopsifoliella* are univoltine. Adults are usually day flying and can be swept from the hosts, but rarely come to light. Several specimens were taken in Malaise traps (DNA barcoded material) and in several cases provide the only accurate phenology data for adults.

#### Distribution.

North America; DNA barcodes suggest a rich fauna in Central America: Mexico, Honduras, Costa Rica, likely also elsewhere in the Neotropics (see under Composition).

#### Etymology.

The name *Aspilanta* is an anagram of *Antispila*, where one “i” was replaced by an “a”. The gender of the name is to be regarded as feminine.

#### Composition.

In the checklist below we provide the original genus in brackets, type locality, and the hostplant of the types. The species are listed according to the position in the recent phylogenetic analyses (Milla et al. 2017, 2019).

We also include the candidate species *Aspilanta* “Vitis1_USA” (van Nieukerken et al. 2012) and *A.* “Vitis.arizonica_USA”. Some publicly available DNA barcodes (Fig. 44) closely match confirmed *Aspilanta* sequences, suggesting further candidate species and a rich fauna in Mexico, Honduras and Costa Rica, but until these taxa are examined morphologically, we omit them. The following BINs are concerned: Mexico: BOLD:ACZ5051, BOLD:ACP0240, BOLD:ACO9420, BOLD:ACU0821, BOLD:ACT4781; Costa Rica: BOLD:ADA1988, BOLD:ACL9188; Honduras: BOLD:ACF9350.

The Neotropic species *Antispila trypherantis* Meyrick, 1916 (Guyana), *A. pentalitha* Meyrick, 1916 (Guyana) and *A. cyclosema* Meyrick, 1921 (Brazil) may also belong in *Aspilanta*, based on their original descriptions that cite the presence of an apical dot, but without examination of types, we refrain from recombination here. Also the Patagonian group of species, associated with *Nothofagus* (Nothofagaceae), for which Nielsen in his unpublished thesis proposed the name “*Neospila*”, could belong in *Aspilanta* on the basis of the very similar externals (Fig. 7), although following the latest phylogeny, where it occurs as Genus14, its inclusion would make *Aspilanta* paraphyletic (Milla et al. 2019) (Fig. 43).

In the Museum of Comparative Zoology (Cambridge, MA), there is a series of externally similar moths of unknown provenance, ex coll. Dietz, allegedly reared from poison ivy, (*Toxicodendron radicans*, Anacardiaceae) with the manuscript name “*Antispila rhoifoliella*” (handwritten label: *on Rhus radicans. Coll 9.7.[18]99, many mines empty, larva pale, green frass line; head + 1 dark brown. [word crossed out] mine begins with a fine tract along edge of leaf, expands & frass collects along edge of mine*.). These specimens have emergence dates of 1–20 June 1900 (written as “19C”). As we have never seen such mines on poison ivy we cannot exclude the possibility that Virginia creeper was mistaken for poison ivy, as both often grow together; in this case the series would likely represent *Aspilanta ampelopsifoliella*. We thus ignore this information until these specimens have been examined in more detail.

### Key to the Nearctic genera of Heliozelidae

**Table:** 

1	Forewing colour pattern comprising silvery white spots and bands on a dark background	**2**
–	Forewing basally uniform silvery to leaden grey (exceptionally with paler longitudinal streaks), distal part comprising a pattern of a yellow to orange background, with three silver strigulae and various amounts of black	*** Coptodisca ***
2	Forewing with an apical silver spot in addition to a fascia at ¼ and two opposite spots at 2/3	***Aspilanta*** (most species)
–	Forewing without apical spot	**3**
3	Forewing with a pattern of a fascia at ¼ and two opposite spots at 2/3	**4**
–	Forewing with only two spots on dorsum, or a spot at ¼ and a narrow fascia at 2/3	*** Heliozela ***
4	Antenna with white tip	***Aspilanta viticordifoliella*** [note: there may be an *Antispila* species with a similar white tip (see under *A. viticordifoliella*). In that case checking wing venation is necessary for the correct identification]
–	Antenna without white tip	*** Antispila ***

### Checklist

*Aspilanta oinophylla* (van Nieukerken & Wagner, 2012): 38, **comb n.** (*Antispila*) USA, Georgia, Murray Co., Vitis aestivalis Michx. var. aestivalis [type species]

*Aspilanta hydrangaeella* (Chambers, 1874a): 170, **comb n.** (*Antispila*) USA, Kentucky, Covington, *Hydrangea arborescens* L.

*Aspilanta ampelopsifoliella* (Chambers, 1874a): 168, **comb n.** (*Antispila*) USA, Kentucky, Covington, *Parthenocissus quinquefolia* (L.) Planchon

*Aspilanta voraginella* (Braun, 1927b): 191, **comb n.** (*Antispila*) USA, Utah, Washington Co., *Vitis arizonica* Engelm.

*Aspilanta argentifera* (Braun, 1927a): 56, **comb n.** (*Antispila*) Canada, Ontario, Sparrow Lake., collected as adult without definite host association.

*Aspilanta viticordifoliella* (Clemens, 1860): 209, **comb n.** (*Antispila*) USA, Pennsylvania, Easton, *Vitis vulpina* L.

### Candidate species

*Aspilanta* “Vitis1_USA” van Nieukerken & Wagner, 2012 (in *Antispila*) USA, Florida, Connecticut, *Vitis aestivalis* Michx.

*Aspilanta* “Vitis.arizonica_USA” USA, Arizona, *Vitis arizonica* Engelm.

### Comparative morphology of Heliozelidae

In order to judge the validity of the morphological characters in *Aspilanta*, they should be compared with the same characters in other Heliozelidae. There is no single large treatment of the family, but Davis (1998) provides an introductory description and a lot of information is found in Nielsen’s unpublished thesis (Nielsen 1980). However, there are very detailed monographs on all stages of species in the genera *Heliozela* (Prota 1962), *Antispila* (Dziurzyński 1948, 1952) and especially for immatures in *Holocacista* (Marchi 1956). For the last genus, the recent taxonomic paper provides further information (van Nieukerken and Geertsema 2015) and for *Coptodisca* a number of papers deal with various aspects (Snodgrass 1922; Peterson 1948; Maier 1988; Bernardo et al. 2015). Some information is also provided in the paper describing the genus *Plesiozela* (Karsholt and Kristensen 2003), but as that genus has now been shown to belong to Incurvariidae (Milla et al. 2019), it is only partly relevant. Unfortunately, there is currently little published information on the detailed morphology of the large Australian clades (*Hoplophanes* group, *Pseliastes* group, Milla et al. 2019), with only a brief adult description of *Hoplophanes* Meyrick, 1897, available in Nielsen’s thesis.

**Adult.***Head*. All Heliozelidae share the smooth head, with appressed, lamellar scales. This is a strong apomorphy for the family. Antennae in *Hoplophanes* have ca. 28–30 flagellomeres, *Heliozela* 15–25, ca. 23 in *Antispila*, 18 in *Antispilina*, 12–18 in *Holocacista*, 12–16 in *Coptodisca* and 14–18 in *Aspilanta*. The flagellomeres are much longer than wide in *Aspilanta* and other genera in the *Holocacista group*, almost the same in *Antispila*, but in *Heliozela* hardly longer than wide; all flagellomeres have two rings of scales. Mandibles are small, but visible in all genera (according to Nielsen absent in the leafmining genera). The proboscis (galea) is more than twice the height of the head in *Heliozela* and *Antispila*, almost twice the height in *Aspilanta*, but hardly longer than the head in *Holocacista, Antispilina* and *Coptodisca*. The maxillary palpi are progressively reduced, from four segments in *Hoplophanes* and *Heliozela*, through three segments in *Antispila* and a reduction in the *Holocacista group* genera into a single segment (Nielsen considered them absent, but we clearly observed a short palp). Labial palpi usually have three segments, but only two in *Antispilina ludwigi*; the last segment is very long in *Aspilanta* (3 × second segment) compared to *Coptodisca* (slightly longer than second segment). Eyes are rather similar in most Heliozelidae, but comparatively large in *Hoplophanes* (interocular index 1.06, see (Davis 1975)), in other genera much smaller (less than 0.6), but in *Aspilanta* larger than in related genera.

*Thorax*. The most relevant character in the legs is the presence of an epiphysis on the foretibia, a plesiomorphic condition in Lepidoptera. It is present in *Hoplophanes*, *Tyriozela*, and *Heliozela*, probably also in the other Australian genera, but absent in *Antispila* and most of the genera in the *Holocacista group*, but *Holocacista rivillei* and *H. capensis* have a reduced epiphysis (van Nieukerken and Geertsema 2015).

Forewings in the majority of Heliozelidae are metallic shining. A forewing pattern of spots and fasciae is present in many Heliozelidae, in *Antispila*, *Antispilina*, *Holocacista* and *Aspilanta* comprising a fascia (or two spots) at one third and an opposite pair of spots at two thirds, somewhat reduced in *Ischnocanaba*. An apical dot is characteristic for most *Aspilanta* species, but something similar also occurs in *Ischnocanaba* and Nielsen’s “*Neospila*”. Larger terminal spots are known in some *Antispila* species. Most *Heliozela* species have the silver spots confined to the dorsal margin, but in some cases one of these reaches the costa, or an extra spot is present on the costa, and a few have a pattern similar to *Antispila*. Such species were previously incorrectly placed in *Antispila* (e.g., *H. anna* (Fletcher, 1920)), like many of the species in *Holocacista* and *Aspilanta*. *Coptodisca* species have a completely different pattern, resembling that of *Leucoptera* (Lyonetiidae).

Androconial scales on one or both wings are common in *Antispila*, but absent from most other genera.

**Figures 9–11.**
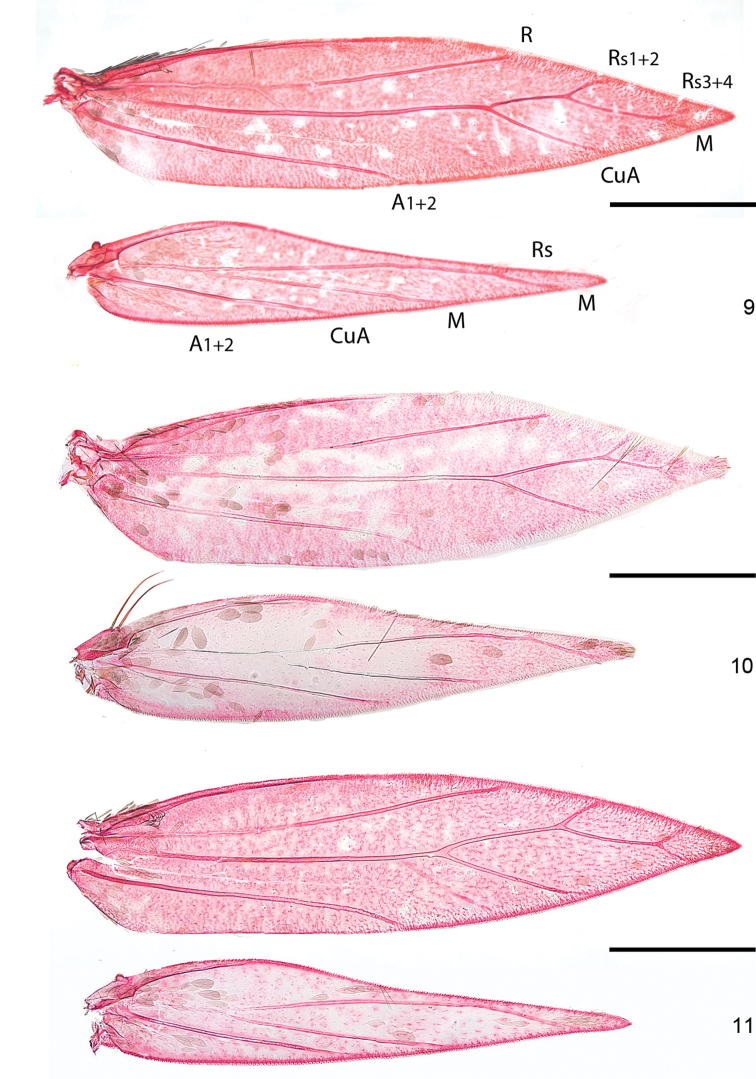
*Aspilanta* species, denuded wings, showing venation. **9***A. oinophylla*, male, veins labelled, RMNH.INS.24257 **10***A. hydrangaeella*, female, RMNH.INS.25191 **11***A. ampelopsifoliella*, male, RMNH.INS.24376. Scale bars: 500 μm.

*Venation*. *Hoplophanes*, *Heliozela*, and *Antispila* have the more complete venation, with a closed cell between Rs and CuA, three branches of Rs; in *Antispilina, Holocacista, Aspilanta* and *Coptodisca* the venation is reduced, Rs, M and Cu are coalescent from wing base, closing the cell, and ending in three or four branches, rarely two. The label CuA given for the *A. oinophylla* forewing by van Nieukerken et al. (2012: fig. 6) is misplaced; the line next to it is a staining artefact. What is labelled M2+3 should be CuA (see Fig. 9). The hindwing of all Heliozelidae lacks the cross-vein M-CuA that is present in most other Adeloidea. The wing coupling comprises in the hindwing a single frenulum in males and 2–5 frenular bristles in females; in *Heliozela sericiella* it is 4–5 according to Prota (1962), but we observe only two in *H. hammoniella*, in Antispila three, in *Holocacista*, *Aspilanta* and *Coptodisca* only two. The forewing retinaculum comprises a distinct cuticular lobe in male *Hoplophanes*, *Tyriozela* and *Heliozela*, provided with ca. four or five hooked scales; the female has a row of setae inserted on the vein Rs+M. In *Antispila* the Sc forms a long fold with a row of scales, female as in *Heliozela*. Males in the *Holocacista group* have a series of hooked scales on the Sc fold, very similar to that structure in Nepticulidae (van Nieukerken 1986).

*Male genitalia*. These are rather uniform throughout Heliozelidae, with a long and narrow vinculum; bilobed or truncate tegumen (sometimes termed uncus); triangular or elongate valvae, each with a single pectinifer with 8–30 blunt sensilla, often different numbers on each valva; a spear-shaped juxta; phallus very long and narrow, with anellar spines and often large projections at phallotrema. Whereas the male genitalia are diagnostic at species level, few characters can be used at higher phylogenetic level. The recurved appendix of the phallus is probably characteristic for *Holocacista*, whereas the very long vinculum (more than twice the valva length) separates most species of the *Holocacista group* from *Antispila*.

*Female genitalia*. The oviscapt is spear shaped with many small teeth in *Hoplophanes*, *Tyriozela* and *Heliozela*, whereas in *Antispila* and the *Holocacista group* it has just a few cusps at either side. In *Antispila* there is a distinct interapodemal process between the anterior apophyses, running almost to the caudal tip. In the *Holocacista* group the process is very short, the caudal part being absent.

**Larva**. Larvae of the Australian genera are unfortunately hardly known. Leafmining larvae of *Heliozela sericiella* (Prota 1962), *Holocacista rivillei* (Marchi 1956) and *Antispila treitschkiella* (Dziurzyński 1948) have been studied in detail, including all instars, and the fourth instar of *A. metallella* by Grandi (1933). The larvae are usually white to yellow or green, flattened and with prognathous head.

*Head*. Fourth instar larvae of *Heliozela* have two pairs of stemmata at either side, those of *Antispila* three, and *Aspilanta* two. In *Holocacista* and *Coptodisca* probably also two, but difficult to see in our slide mounted specimens. Davis (1978, 1998) reports 2–5 for the family.

*Thorax*. Legs are absent in most studied larvae, but some *Heliozela* species have much reduced legs during the 5^th^, non-feeding instar (Prota 1962). Only for full grown *H. aesella* larvae fully developed thoracic legs are reported (McGiffen and Neunzig 1985; Davis 1987), which CSE has also observed in live larvae (including in early instars). At least some of the Australian taxa also have more developed legs, although many are also legless (Andy Young & Mike Halsey, pers. comm.). In most cases the prosternum and protergum are elongate sclerotised structures, in *Coptodisca* sometimes adorned with a ventral transverse row of swellings.

*Abdomen*. Prolegs are only reported from 4^th^ instar *Heliozela* larvae on segments 3–6, with 3–8 crochets in single or sometimes double mesoseries. Instead most larvae show either paired or unpaired calli, the position of which may be diagnostic, but is difficult to assess in slide mounted specimens. The integument is covered by slight, often transverse swellings, which may concentrate in the centre of the segments, and in some cases form a row of abdominal plates in species of *Antispila*. The presence of warts on the 8^th^ larval segment, used for stridulation or drumming (Low 2008; van Nieukerken et al. 2018) is typical for many species of *Antispila*; they are unknown for the other genera.

**Cocoon.** Cocoons (shields) of leafmining species are prepared from both epidermal layers of the final part of the leafmine, held together by silk, and are very similar in most genera. *Antispila* has as extra character in that the larva contracts the case with silk, resulting in a lengthwise keel; also in all *Antispila* species there are silken projections from the anterior (and sometimes the posterior) end of the case; we do not know them from *Heliozela*, *Holocacista* or *Coptodisca*, but some *Aspilanta* species produce these projections at least sometimes (Fig. 106) (Eiseman 2019). Cocoons are made by 4^th^ instar larvae and not by the final instar as some authors state incorrectly. The final moult takes place in the shield.

**Pupa.** The pupa of *Heliozela sericiella* was described by Prota (1962); otherwise there is little published information on pupae of Heliozelidae.

**Phylogeny.** We use the recent molecular phylogeny based on transcriptome data, including up to 1049 nuclear genes (Milla et al. 2019) as the basis for further discussions on the phylogeny. Part of this tree is here reproduced, with the name *Aspilanta* instead of *Antispila* group II, and with the listed apomorphies added (Fig. 43). The resulting tree is in fact not that different from the manually derived tree, based on morphology, in Ebbe Nielsen’s thesis (see Milla et al. 2017). The most apparent differences are the removal of *Plesiozela* from Heliozelidae to Incurvariidae, the position of *Antispilina* (Nielsen placed it next to *Antispila*) and the non-monophyly of the Australian *Hoplophanes* and *Pseliastis*. Also, Nielsen did not study any of the species here placed in *Aspilanta*. Interestingly, the leafmining genus groups, viz. the *Heliozela* group, the genus *Antispila* (= *Antispila group*) and the *Holocacista* group together form a monophyletic group, already recognised by Nielsen (1980). Here we provide possible apomorphies for the genera and clades recognised in this part of the tree only, based on Nielsen’s work and our own observations. Characters without an asterisk were already listed by Nielsen (1980). They are not explained further when treated above.

Possible apomorphies for the *Heliozela*, *Antispila*, and *Holocacista* groups are:

(1) Size strongly reduced. The wingspan of all the species of these genera is small, always less than 9 mm, and usually smaller.

(2) Compound eyes in a posteroventral position. The anterior lateral margin of the compound eyes does not reach the anterior surface of the head capsule, but is present behind the laterally extended genae.

(3) Proboscis very long, two to three times as long as height of head capsule. However, as noted above, it is shorter again in the small moths of *Holocacista*, *Antispilina*, and *Coptodisca*.

Nielsen also listed absence of mandibles as an apomorphy, but we think that reduced mandibles are in fact present.

A possible apomorphy for the *Heliozela* group (including *Tyriozela*) is:

(4) Female with spear-shaped ovipositor tip. The flattened, deeply dented ovipositor observed in the *Antispila* and *Holocacista* groups is regarded as the relatively plesiomorphic state compared to the spear-shaped tip of the *Heliozela* group. The first mentioned type is present in most Incurvariidae and the most generalised genera of Prodoxidae.

Two other characters given by Nielsen are in our view incorrect; the retinaculum is not as long as Sc as he states, and although some species have petiole-mining larvae, this is not the only larval feeding mode; many Australian and Oriental species are leafminers, and others are gall formers.

Possible apomorphies for *Antispila* and the *Holocacista* group are:

(5) Head with transfrontal suture absent and scale-sockets on the entire frons and vertex. The area in all other Adeloidea is strengthened and devoid of scale-sockets.

(6) Foretibia without fully developed epiphysis.

(7) Pseudofrenular bristles absent in male.

(8) Maxillary palpi three-segmented or shorter.

(9) Microtrichiation reduced on forewings. Only the proximal third is provided with microtrichia; in *Hoplophanes* the entire wing surface is covered with microtrichia, what is also regarded as a ground-plan condition within Adeloidea.

(10)*. Larvae without legs and prolegs.

(11)*. Forewing with “*Antispila* pattern” (see above), secondarily derived in *Coptodisca* and a few species of *Holocacista*.

Possible apomorphies for *Antispila* s. str. are:

(12)* Cocoon folded lengthwise, resulting in a distinct keel.

(13)* The presence of warts on the 8^th^ larval segment may be an additional apomorphy, but more likely only for a subset of the genus.

Nielsen gave the presence of an interapodemal process in female as important apomorphy for the *Antispila* group, but we see a similar structure, albeit somewhat reduced, in *Holocacista* (van Nieukerken and Geertsema 2015: figs 53, 68) and *Aspilanta* (see Fig. 29). It is possible that this character is another apomorphy for *Antispila* + *Holocacista* group.

Possible apomorphies for the *Holocacista* group are:

(14). Venation strongly reduced: cell absent in both fore- and hindwings.

(15). Male retinaculum comprising a series of hook-shaped setae.

(16). Sublateral process of the transtilla in male genitalia long and slender.

(17). Larval case attached freely to a branch of the food-plant or other material.

(18)*. Male genitalia: vinculum extremely extended.

(19)*. Number of antennal segments reduced.

(20)*. Reduction of maxillary palps to one segment.

Possible apomorphies for *Antispilina* are:

(21). Larvae mining the leaves of herbaceous Polygonaceae.

(22)*. Labial palpi reduced to 2 segments.

A possible apomorphy for *Holocacista* is:

(23)*. Phallus with long, often recurved appendix.

Possible apomorphies for *Aspilanta* are:

(24)*. Forewing with apical spot.

Possible apomorphies for *Coptodisca* are:

(25). Forewing pattern specialised.

**DNA barcoding.** We provide COI DNA barcode sequences as an aid for identification for all known species of *Aspilanta*. A Neighbor-Joining tree of these (Fig. 44), including some nearest neighbours (NN) and some species of the sister-group *Coptodisca*. Details on the Barcode Identification Number (BIN), the average and maximum distance within each BIN, and the distance to NN are given for each species. All species are well distinguished by barcodes. *Aspilanta hydrangaeella* has two BINs, with a considerable distance, and *A. viticordifoliella* has three BINs, although the specimen with BIN BOLD:ACA6487 has not been studied; its identification is only based on the barcode.

## Treatment of species

### Key to known species of *Aspilanta*

**Table:** 

1	Forewing with an apical silver spot in addition to a fascia at ¼ and two opposite spots at 2/3; antenna dark throughout or with the last 1–3 segments white	**2**
–	Forewing without apical spot; antenna with distinct white tip of 2 or 3 segments	***A. viticordifoliella***
2	Antennae with last 3 segments clearly white. Larvae mine *Hydrangea*	***A. hydrangaeella***
–	Antennae dark throughout, or at most last segment white. Larvae mine Vitaceae or Myricaceae	**3**
3	Head with silvery white scales, forewing with distinct fringe line, fringe almost white. Larvae on Vitaceae	***A. oinophylla, ampelopsifoliella* or “*Vitis1***” See van Nieukerken et al. (2012) for differences in genitalia
–	Head with darker bronze brown or brassy scales, forewing with rather indistinct fringe line, fringe grey brown. Larvae on Vitaceae or Myricaceae	**4**
4	Eastern species (ON & NL to NC), larvae on Myricaceae	***A. argentifera***
–	Southwestern species (AZ, UT, TX), larvae on *Vitis*	***A. voraginella***

#### Aspilanta oinophylla

Taxon classificationAnimaliaLepidopteraHeliozelidae

(van Nieukerken & Wagner, 2012) comb. n.

E4B392D2-AD17-56E5-BF3E-2ABEB1090819

Figs 1 , 9 , 14–20 , 21 , 26 , 30 , 32 , 40 , 42 , 45 , 51–58

Antispila oinophylla van Nieukerken & Wagner, 2012: 38. Holotype ♂, USA: Georgia, Murray Co., Chattahoochee Nat. Forest, E of Chatsworth, GA rd 52, 523 m, 34.74066N, 84.71852W, hardwood forest along highway, leafmines on Vitis aestivalis var. aestivalis, 14.x.2010, EvN2010266, emerged 14.iv–4.v.2011, E.J. van Nieukerken & C. Doorenweerd, Genitalia slide EJvN 4204, RMNH.INS.24204 (RMNH) [examined].
Antispila oinophylla ; Wang et al. 2018: 1 [pheromones]; van Nieukerken and Pohl 2018: 42; Eiseman 2019: 726, 729, 733.
[Antispila ampelopsifoliella; Needham et al. 1928: 289 [partim]; Davis 1983b: 4 [partim]; Grehan et al. 1995: 46 [partim]; Laštůvka 2009: S57; van Nieukerken et al. 2011: 51. Misidentifications.]
[Antispila ampelopsiella; Chambers 1877: 195 [partim]; Chambers 1879: 126 [partim] Dyar et al. 1903: 539 [partim]; Barnes and McDunnough 1917: 181 [partim]; Forbes 1923: 226; McDunnough 1939: 91 [partim]; Brower 1984: 29 [partim]. Misidentifications.]

##### Differential diagnosis.

Wingspan ca. 4.8–6.2 mm, forewing length 2.3–2.8 mm. Externally inseparable from *A. ampelopsifoliella* and *A.* “Vitis1_USA”. The silvery white head separates it from *A. voraginella* and *A. argentifera*, and *A. hydrangaeella* has distinct white antennal tips. Male genitalia with characteristic long curved process and a comb of 10–12 large teeth at phallotrema; tegumen truncate; valvae with 10–12 pecten sensilla. Female oviscapt with more cusps (4–5 at either side) than *A. ampelopsifoliella* (3). Leafmines differ from other Vitaceae miners by compact size, rather short linear portion close to a vein and frass in concentric lines, especially in thin leaves.

##### Host plants.

Vitaceae: *Parthenocissus quinquefolia*, *P. vitacea*, *Vitis aestivalis*, *V. labrusca*, *V. riparia*, *V. vinifera*, *V. vulpina*.

##### Leafmines.

(Figs 51–58) Egg usually within 1–2 mm from a vein. The mine starts as a rather straight or slightly contorted linear mine towards the vein, usually forming a right angle and often following the vein for a short distance, then again turning away from the vein and expanding into a blotch. The linear portion of the mine is usually later incorporated into the blotch. The frass in the linear portion usually occupies the complete mine width, occasionally deposited in a thin line. In the blotch much of the blackish-brown frass is deposited close to the origin in semi-circular concentric frass lines, best seen in thin shade leaves; in sun-exposed leaves the frass pattern is often obscured. The whole mine occupies as a rule an area of less than 10 × 10 mm; only in thin leaves are mines appreciably larger. The larva cuts out an elliptic case ca. 3.2–4.0 mm long.

##### Larva.

Yellowish green, with green gut contents; head and prothorax brown.

##### Life history.

Larvae in Canada and NE United States in July, August and September, more south until October; in Italy in June–July and August–October. Adults from June to August; apparently bivoltine.

##### Distribution.

Canada: Ontario, Quebec; USA: Connecticut, Georgia, Kentucky, Massachusetts*, Minnesota*, New York, North Carolina*, Oklahoma*, Tennessee, Vermont, Wisconsin*; Europe: Italy (introduced).

##### Barcode.

BIN: BOLD:AAI4367 average distance 0.42%, max. distance 1.28% (*n* = 41), distance to nearest neighbour 9.79% (*A. voraginella*).

##### Parasitoids.

Eulophidae: *Cirrospilus pictus* (Nees, 1834) (Italy) (BOLD:ACZ7659), *Cirrospilus* sensu lato, including *Burkseus* (see Perry and Heraty 2019) (OK), Pediobius ? albipes (Provancher, 1887) (NC); Braconidae: *Gnamptodon* sp. (NY) (BOLD:ACZ9790), *Mirax* sp. (VT) (BOLD:ACZ7174).

##### Remarks.

In the original description it was noted that the species had not been found on *Parthenocissus* in North America. Since then some specimens of this species were barcoded and reared from *Parthenocissus* by J.-F. Landry (material in CNC), in a situation where *Vitis* and *Parthenocissus* grew entangled. Even though this would weaken the argumentation about the identity of *A. ampelopsifoliella* by van Nieukerken et al. (2012), we observe that still the large majority of mines on *Parthenocissus* in North America that we have seen belong to other species. Selection of a Neotype for *A. ampelopsifoliella* should settle this nomenclatorial issue (see below).

##### Material.

**Adults examined.** CANADA – **Ontario** • 2; Normandale; 42.71N, 80.31W; Freeman & Lewis leg.; *Vitis*; emerged 22–25 Mar. 1957; EventId: 56–168; CNCLEP00122325–00122326. • 2; Normandale; 42.71N, 80.31W; Freeman & Lewis leg.; *Vitis*; emerged 19–25 Feb. 1960; EventId: 59–198; CNCLEP00122327–00122328. • 1 ♂; Ottawa; 45.41N, 75.69W; G.G.Lewis leg.; *Vitis*; emerged 25 Feb. 1971; EventId: 70–53; Genitalia slide: MIC1871; CNCLEP00100104. • 1 ♀ 1; Ottawa; 45.41N, 75.69W; G.G.Lewis leg.; *Parthenocissus*; emerged 15, 29 Mar. 1971; EventId: 70–48; Genitalia slide: MIC1877; CNCLEP00100105–00100106. • 1 ♂1 ♀ 3; Ottawa; 45.41N, 75.69W; Freeman, Lewis leg.; *Vitis*; emerged 21 Mar. – 08 Apr. 1958; EventId: 57–112; Genitalia slide: MIC1872, MIC1873; CNCLEP00100467–00100468, 00122312–00122314. • 1 ♂ 1; Ottawa; 45.41N, 75.69W; T.N. Freeman leg.; *Vitis*; emerged 16–18 Apr. 1957; EventId: 56–183; Genitalia slide: MIC1875; CNCLEP00122315, 00122316. • 1 ♂ 6; Overbrook; 45.42N, 75.65W; G.G. Lewis leg.; *Vitis*; emerged 15–24 Aug. 1955, 04–06 Jul. 1956; EventId: 55–53; Genitalia slide: MIC1874, MIC1878; CNCLEP00122317 – 00122324. – **Québec** • 1; Gatineau, Aylmer, 48 rue du Couvent; 45.3967N, 75.849W; alt. 80 m; 14 Jul. 2010; J.-F. Landry leg.; *Vitis riparia*; CNCLEP00097660. • 3; same data as preceding; emerged 31 Aug. – 02 Sep. 2011; CNCLEP00091691–00091693. • 4; same data as preceding; *Parthenocissus quinquefolia*; emerged 01–02 Sep. 2011; CNCLEP00091694– 00091697. • 10; same data as preceding; alt. 80 m; J.-F. Landry leg.; *Vitis riparia*; emerged 31 Aug. 2011; CNCLEP00097700–00097709. • 1; Hull; 45.435N, 75.708W; T.N. Freeman leg.; *Vitis*; emerged 07 Jul. 1956; EventId: 55–228; CNCLEP00122305. • 3; Québec, Hull; 45.435N, 75.708W; T.N. Freeman leg.; *Vitis*; emerged 01–07 Jul. 1956; EventId: 55–228; CNCLEP00122306–00122308. • 2; Hull; 45.435N, 75.708W; G.G. Lewis leg.; *Vitis*; emerged 12–15 Aug. 1955; EventId: 55–60; CNCLEP00122309–00122310. • 1; Québec, Hull; 45.435N, 75.708W; G.G. Lewis leg.; *Vitis*; emerged 30 Jun. 1956; EventId: 55–60; CNCLEP00122311.

USA – **Oklahoma** • 1; Payne Co., Mehan; 36.014339N, 96.996744W; 10 Jul. 2016; Michael W. Palmer leg.; *Vitis*; emerged 10 Aug. 2016; EventId: CSE2971; CSEC. – **Vermont** • 1 ♂, 9; Addison Co, Button Bay SP, Lake Champlain borders; 44.18154N, 73.36892W; alt. 40–50 m; 16 Sep. 2011; E.J. van Nieukerken leg.; *Vitis riparia*; emerged 21 May–05 Jun. 2012; EventId: EvN no 2011253–1K; Genitalia slide: EvN4378; RMNH.INS.24378; RMNH.

##### Larvae and leafmines examined.

CANADA – **Ontario** • leafmines and larvae, rearing failed; Chatham–Kent Div., Rondeau Prov. Park, Campground; 42.3223N, 81.8438W; alt. 177 m; 24–25 Jul. 2015; E.J. van Nieukerken leg.; *Vitis riparia*; EventId: EvN no 2015091–1K; RMNH.INS.40130. • Haldimand Co., Dunnville, along Highway 3; 42.91708N, 79.58009W; alt. 180 m; 19 Jul. 2015; E.J. van Nieukerken leg.; *Vitis riparia*; EventId: EvN no 2015068–H/ EvN no 2015068–K; RMNH.INS.40089–40090. • Norfolk Co., Long Point Prov. Park, Cottonwood campground; 42.58039N, 80.4085W; alt. 174 m; 20 Jul. 2015; E.J. van Nieukerken leg.; *Vitis riparia*; EventId: EvN no 2015069–H/2015069–K; RMNH.INS.40091–40092. • Ottawa, Hintonburg, Bayview Rd; 45.40878N, 75.72459W; alt. 58 m; 12 Jul. 2018; E.J. van Nieukerken leg.; *Vitis riparia*; EventId: EvN no 2018081–H; RMNH.INS.46213. • Ottawa, Hintonburg, Fairmont Ave; 45.39979N, 75.72046W; alt. 67 m; 08 Sep. 2015; E.J. van Nieukerken leg.; *Vitis riparia*; EventId: EvN no 2015232–K; RMNH.INS.40384. • 3 larvae (used for transcriptome studies), leafmines; Ottawa, Parkdale, along Ottawa river; 45.41184N, 75.73369W; alt. 55 m; 13 Sep. 2015; E.J. van Nieukerken leg.; *Vitis riparia*; EventId: EvN no 2015246–M/2015246–K; RMNH.INS.30596–30598, 40403. • Ottawa, Parkdale, along Ottawa river; 45.41216N, 75.73006W; alt. 55 m; 13 Sep. 2015; E.J. van Nieukerken leg.; *Vitis riparia*; EventId: EvN no 2015245–K; RMNH.INS.40402. – **Québec** • Gatineau, Aylmer E, near Ottawa river; 45.39261N, 75.78704W; alt. 56 m; 12 Sep. 2015; E.J. van Nieukerken leg.; *Vitis riparia*; EventId: EvN no 2015235–K; RMNH.INS.40387.

USA – **Massachusetts** • Franklin Co., Northfield; 42.646762N, 72.42527W; 13 Sep. 2016; Charley Eiseman leg.; *Vitis aestivalis*; CSEC.

##### BOLD data, material not examined.

CANADA – **Ontario** • 3; Grand Bend, Pinery Provincial Park, Site 2; 43.2699N, 81.8271W; alt. 178 m; 25 Jun.–09 Jul. 2014; CBG Collections Staff leg.; EventId: GMP#03351; BIOUG33534–B01, BIOUG33534–C01, BIOUG33534–D01. • 1; Point Pelee National Park, Cactus Field; 41.939N, 82.516W; alt. 168 m; 27 Jun.–04 Jul. 2012; Tyler Peters leg.; EventId: GMP#00175; BIOUG03514–B07. • 1; Wellington County, Puslinch Township, Concession 11/Hume Rd; 43.537N, 80.134W; alt. 320 m; 10–17 Jul. 2010; Paul Hebert leg.; EventId: L#PHPUS–016; BIOUG02834–E04. • 1; Lambton Co., Port Franks; 43.22N, 81.91W; 02 Jul. 2017; K. H. Stead leg.; EventId: KSLEP1081–17; KSLEP1081–17; Research Collection of Ken Stead. • 1; Lambton Co., Port Franks; 43.2257N, 81.916W; alt. 188 m; 30 Jul. 2012; K.H.Stead leg.; BIOUG20646–E04. • 1; same locality data as preceding; 02 Aug. 2012; K.H.Stead leg.; BIOUG16764–D05. – **Québec** • 1; Montreal, Montreal Botanical Garden, Trap 2; 45.5594N, 73.5668W; alt. 52 m; 11–18 Jun. 2014; Maxim Larrivee leg.; EventId: GMP#04699; BIOUG25652–B07.

ITALY – **South Tyrol** • 1; Bolzano, Kaltern/ Altenburger Wald, Umg. Ziegelstadel; 46.379N, 11.229E; alt. 705 m; 10 Aug. 2015; Huemer P. leg.; TLMF Lep 18731; TLMF.

##### Observations.

ITALY (all from http://www.lepiforum.de/lepiwiki.pl?Antispila_Oinophylla) – **South Tyrol** • 1 adult; Bolzano, Bozen–Rentsch; 46.502N, 11.366E; alt. 265 m; 08 Aug. 2014; Werner Pichler leg.. • many adults; South Tyrol, Bolzano, Bozen–St. Magdalena; 46.503N, 11.372E; alt. 250–600 m; 01 Jul.–30 Aug. 2015; Werner Pichler leg.; *Vitis vinifera*. • Bolzano, Eisacktal, Klausen; 46.64N, 11.56E; alt. 650 m; 09–11 Jul. 2019; Dieter Robrecht leg.; *Parthenocissus vitacea*; Robrecht, Dieter, personal collection. • 15 adults; South Tyrol, Bolzano, Eisacktal, Klausen [Chiusa]; 46.64N, 11.56E; alt. 650 m; 09–11 Jul. 2019; Dieter Robrecht leg.; *Vitis vinifera*; emerged 01–06 Aug. 2019; Robrecht, Dieter, personal collection.

USA – **Minnesota** • Wabasha Co., Weaver Dunes Preserve; 44.258N, 91.932W; 17 Jul. 2015; Charley Eiseman & Julia Blyth leg.; *Vitis*; https://www.inaturalist.org/observations/44819628. – **North Carolina** • Durham Co., 17–acre wood preserve; 36.024N, 78.925W; 29 Sep. 2017; Tracy Feldman leg.; *Vitis*; EventId: CSE4561; https://bugguide.net/node/view/1447964. – **Wisconsin** • Dane Co., Cross Plains; 43.12N, 89.66W; 19 Sep. 2011; Ilona Loser leg.; *Vitis riparia*; https://bugguide.net/node/view/578758/bgimage.

#### Aspilanta hydrangaeella

Taxon classificationAnimaliaLepidopteraHeliozelidae

(Chambers, 1874) comb. n.

13C3CFAD-89D3-5B0E-835D-F0A511550FBD

Figs 2 , 10 , 27 , 28 , 46 , 59–68

Antispila hydrangaeella Chambers, 1874a: 170. Syntypes leafmines and larvae: [USA: Kentucky, Covington] on Hydrangea nivea [probably lost].
Antispila hydrangiaeella Chambers, 1878: 113. Subsequent incorrect spelling.
Antispila hydrangaeella ; Chambers 1877: 195; Chambers 1879: 126; Edwards 1889: 126; Davis 1983b: 4; van Nieukerken et al. 2012: 56; Eiseman 2019: 190, 1333.
Antispila hydrangiaeella ; Dyar et al. 1903: 539; Forbes 1923: 226; Needham et al. 1928: 290; McDunnough 1939: 91.

##### Differential diagnosis.

Wingspan ca. 5.0–5.8 mm, forewing length 2.2–2.8 mm. Externally easily separable from other *Aspilanta* species with terminal spot by the white terminal antennal segments (3 flagellomeres with 6 scale rings). Male genitalia characterised by the two long curved terminal spines at phallotrema, the bearded setae on the juxta, the larger number of sensilla (ca 17–21) of the valval pecten and the bilobed tegumen. Female genitalia not examined.

**Figures 12–13.**
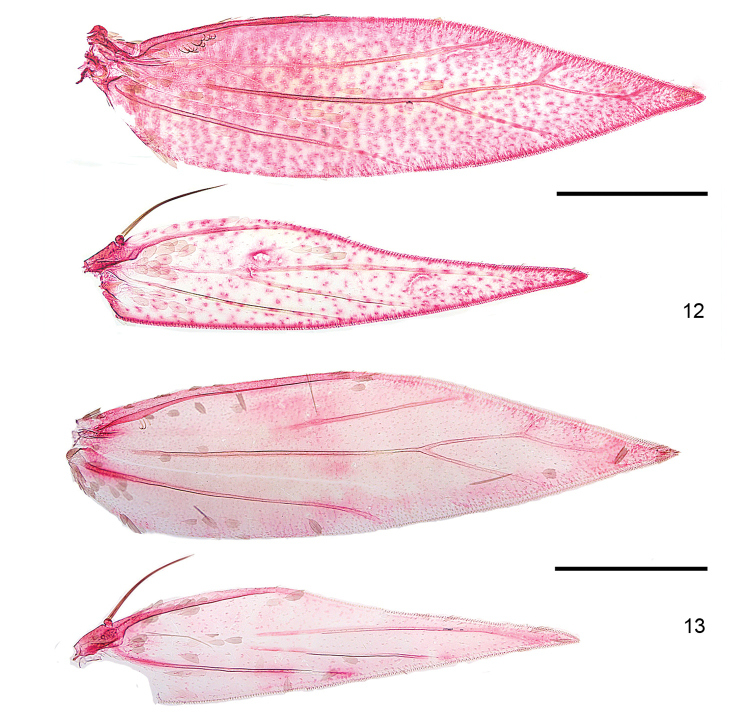
*Aspilanta* species, denuded wings, showing venation. **12***A. voraginella*, male, RMNH.INS.23917 **13***A. argentifera*, male, RMNH.INS.23917. Scale bars: 500 μm.

**Figures 14–20.**
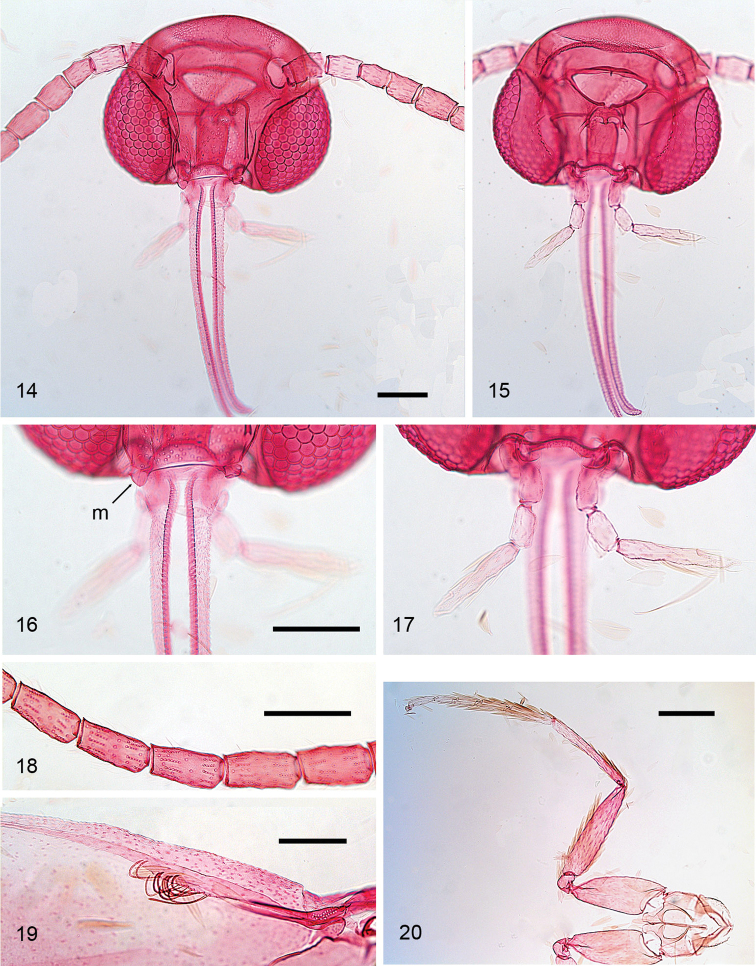
*Aspilanta oinophylla*, adult (male) morphology, RMNH.INS.24440. **14, 15** Head, anterior and posterior views **16, 17** Detail of mouthparts, anterior and posterior views; m = mandible **18** Detail of flagellomeres, middle part of antenna, showing scale sockets of two scale rings per flagellomere **19** Forewing, underside, detail of retinaculum **20** Prothorax with forelegs. Scale bars: 100 μm, 200 μm (**20**).

##### Host plant.

Hydrangeaceae: *Hydrangea arborescens*. Chambers (1874a) recorded it from “wild *Hydrangea* (*H. nivea*)”, and later as *H. radiata* (Chambers 1878), the correct name for that species. However, according to current knowledge, *H. radiata* occurs only in the southern Appalachians (Kartesz and Biota of North America Program (BONAP) 2015; Freeman 2016), and not in Kentucky, so we assume that Chambers also found the mines on *H. arborescens*. To be searched for on *H. cinerea* and *H. radiata*.

##### Leafmines.

(Figs 59–68) Egg inserted anywhere on the leaf, but frequently near a vein. Mine starts with a rather long linear portion, which may be partly or completely contorted, or often follows veins for some length; later the mine turns into an elongate blotch or a wide gallery. Frass starts usually as a rather narrow line, often broken and irregular, not always in the middle, later becoming more dispersed, in grains or sometimes smeared out, green to brown; in the blotch the frass remains in the middle, smeared out, dark green to black. Often many mines occur in the same leaf; mines can be rather extensive in the thin leaves of *Hydrangea*. The larva cuts out an elliptic case ca. 3.5–4.5 mm long.

##### Larva.

Colourless or whitish except for green gut contents; head and prothorax dark brown, some darker dots on most segments visible (Fig. 68).

##### Life history.

Poorly known; larvae found in June, August, September and October; adults emerged the following spring from larvae collected in October, but dates are too early due to forced emergence. Chambers provides no information whatsoever; Forbes (1923) reports moths in August. This, together with an early mine photographed in Kentucky on 30 June, suggests the species may be bivoltine.

##### Distribution.

USA: Georgia, Illinois, Kentucky, Maryland*, North Carolina, Ohio*, Tennessee*.

##### Barcode.

Georgia and Ohio population: BIN: BOLD:AAV5059 (*n* = 2, average distance = 2.15%), Tennessee population: BOLD:AAV5060 (*n* = 1), distance between these nearest neighbours 5.25%.

##### Parasitoids.

Eulophidae: *Pediobius albipes* (Provancher, 1887) (GA) (BOLD:ACZ8030), *P. ocellatus* Peck, 1985 (GA, NC) (BOLD:ACZ8031); Braconidae: Microgastrinae sp. (GA) (BOLD:ADA0313).

##### Remarks.

In our barcode analysis we found two clusters with a distance of 5.25%, even the distance of two specimens of the Georgia population is rather large with 2.15%. By analysing the characters of adults of the two barcoded populations, we did not find any supporting argument for the earlier suggestion that two species might be involved (van Nieukerken et al. 2012). Mines of the Georgia and Ohio populations seem to have more contorted mines than the population from North Carolina, but there is also variation in this character within these populations. A further analysis of material is needed to assess this situation, and designation of a Neotype from Kentucky might be necessary to avoid confusion if the species appears to be part of a complex.

##### Material: Adults examined.

USA – **Tennessee** • 1; Blount Co., NP Great Smoky Mts, Lead Cove Trail; 35.59976N, 83.73998W; alt. 690 m; 03 Oct. 2010; E.J. van Nieukerken & C. Doorenweerd leg.; *Hydrangea arborescens*; emerged 04 Apr. 2011; EventId: EvN no 2010141–K; RMNH.

##### Larvae and leafmines examined.

USA – **Ohio** • 2 larvae, Hocking Co., South Bloomingville, Deep Woods Farm; 39.406165N, 82.574946W; alt. 229 m; 13 Sep. 2012; C. Eiseman leg.; *Hydrangea arborescens*; EventId: CSE-OH; RMNH.INS.29601.P, RMNH.INS.29602.P. – **Tennessee** • leafmines; Blount Co., NP Great Smoky Mts, Lead Cove Trail; 35.59976N, 83.73998W; alt. 690 m; 03 Oct. 2010; E.J. van Nieukerken & C. Doorenweerd leg.; *Hydrangea arborescens*; EventId: EvN no 2010141–K; RMNH.INS.43100.

##### Observations.

USA – **Georgia** • Gilmer Co., Ellijay; 34.651N, 84.608W; 23 Sep. 2019; Lisa Kimmerling leg.; *Hydrangea arborescens*; https://www.inaturalist.org/observations/33268524. – **Kentucky** • Bullitt Co., Bernheim Arboretum and Research Forest; 37.913N, 85.648W; 30 June 2019; Mike Plagens leg.; *Hydrangea arborescens*; https://www.inaturalist.org/observations/29522629. – **Maryland** • Harford Co., Susquehanna State Park; 39.605N, 76.152W; 11 Aug. 2018; Josh Emm leg.; *Hydrangea arborescens*; https://bugguide.net/node/view/1573751/bgimage. – **North Carolina** • Madison Co., Along Appalachian Trail southbound from Devil’s Fork Gap; 36.0082N, 82.6098W; 05 Oct. 2019; Jim Petranka leg.; *Hydrangea arborescens*; https://bugguide.net/node/view/1736188/bgimage. – **Ohio** • Hocking Co., Rockbridge, Crane Hollow Preserve; 39.48N, 82.584W; 05 Aug. 2016; Charley Eiseman & Julia Blyth leg.; *Hydrangea arborescens*; https://www.inaturalist.org/observations/44820974.

#### Aspilanta ampelopsifoliella

Taxon classificationAnimaliaLepidopteraHeliozelidae

(Chambers, 1874) comb. n.

652B223F-3598-55D5-9DAF-DC089BCF8D0E

Figs 3 , 11 , 22 , 33 , 34 , 37–39 , 47 , 48 , 69–79

Antispila ampelopsifoliella Chambers, 1874a: 168. Syntypes: leafmines [USA: Kentucky, Covington] on Ampelopsis quinquefolia [= Parthenocissus quinquefolia], “pseudotypes”, Kentucky, Covington (MCZ) [examined].
Antispila ampelopsisella Chambers, 1874b: 197. Subsequent incorrect spelling.
Antispila ampelopsiella Chambers, 1874b: 198. Subsequent incorrect spelling.
Antispila ampelopsilofoliella : internet error.
Antispila ampelopsifoliella ; Edwards 1889: 126; Needham et al. 1928: 289 [partim]; Davis 1983b: 4 [partim]; Grehan et al. 1995: 46 [partim]; van Nieukerken et al. 2012: 54; van Nieukerken and Pohl 2018: 41; Eiseman 2019: 190, 434, 729, 730.
Antispila ampelopsiella ; Chambers 1877: 195 [partim]; Chambers 1879: 126 [partim]; Dyar et al. 1903: 539 [partim]; Barnes and McDunnough 1917: 181 [partim]; Forbes 1923: 226; McDunnough 1939: 91 [partim]; Brower 1984: 29 [partim].

##### Differential diagnosis.

Wingspan ca. 5.0–5.3 mm, forewing length 2.4–2.6 mm. Externally inseparable from *A. oinophylla* (see above) and *A.* “Vitis1_USA”. Male genitalia characterised by the non-bilobed tegumen, pecten with 11–14 sensilla, and phallus with rather small curved spine at phallotrema. Female oviscapt with 3 cusps at either side. Leafmines recognised by long linear portion that is often sinuous.

##### Host plants.

Vitaceae: *Parthenocissus quinquefolia*, *P. vitacea*.

##### Leafmines.

(Figs 69–79) Egg in the majority of examined mines close to midrib, when at leaf margin then usually close to leaf base, occasionally near another vein. The mine starts with a long linear portion, often very sinuous, occasionally straighter, when it runs along the leaflet margin; gradually widening into elongate blotch. Frass brown to black, initially broken linear, later irregularly dispersed, not always in centre of linear portion; in blotch more or less in centre throughout. The larva cuts out an elliptic case ca. 3.5–4 mm long.

**Figures 21–28.**
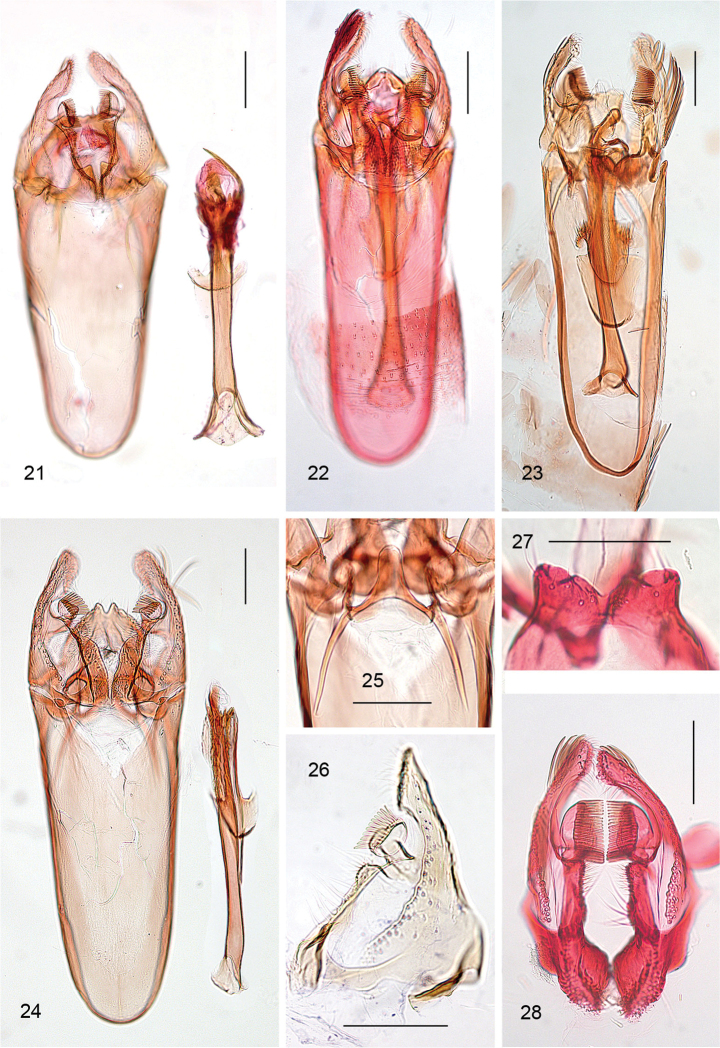
*Aspilanta* species, male genitalia. **21***A. oinophylla*, phallus separate, RMNH.INS.23920 **22***A. ampelopsifoliella*, phallus in situ, RMNH.INS.24376 **23***A. hydrangaeella*, phallus in situ, RMNH.INS.25192 **24***A. voraginella*, phallus separate, holotype, slide EJvN 3916 **25** ditto, detail of transtilla **26***A. oinophylla*, detail valva (flattened), RMNH.INS.15247 **27, 28***A. hydrangaeella*, RMNH.INS.25190, details of resp. tegumen and valvae. Scale bars: 100 μm.

**Figures 29–33.**
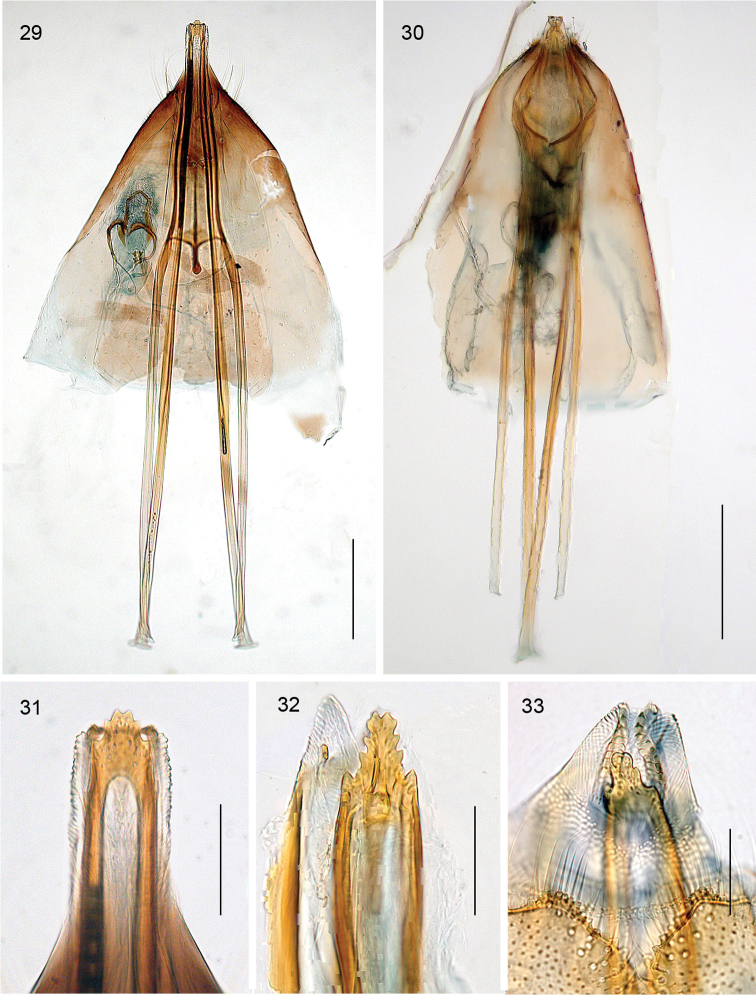
*Aspilanta* species, female genitalia. **29***A. argentifera*, RMNH.INS.25019 **30***A. oinophylla*, RMNH.INS.24211. **31–33** Tip of oviscapt: **31***A. argentifera*, RMNH.INS.25019 **32***A. oinophylla*, RMNH.INS.24211 **33***A. ampelopsifoliella*, RMNH.INS.15220. Scale bars: 200 μm (**29, 30**); 50 μm (**31–33**).

##### Larva.

Colourless or whitish except for green gut contents, in contrast with yellow-green larva of *A. viticordifoliella* on the same host plant (compare Figs 69, 70 with 97–100); head and prothorax almost black.

##### Life history.

Poorly known. Larvae found from June (Oklahoma), August and September, field caught adults only from early July. The adult from the June larva emerged the following spring, like those from August and September larvae, suggesting that this species is univoltine.

##### Distribution.

Canada: Ontario; USA: Connecticut, Kentucky, Massachusetts*, New York, Oklahoma*, Rhode Island*, Vermont, Wisconsin*.

##### Barcode.

BIN: BOLD:ABA3237, average distance 0.3%, max. distance 1.12% (*n* = 10), distance to nearest neighbour 8.79% (BOLD:ACO9420, an unidentified Heliozelidae from Mexico, most likely an *Aspilanta* species).

##### Parasitoids.

Eulophidae: *Chrysocharis paradoxa* Hansson, 1985 (MA), *Closterocerus cinctipennis* Ashmead, 1888 (MA); Braconidae: Microgastrinae sp. (MA), *Pseudognaptodon* sp. (VT).

##### Remarks.

The identity of this species follows from the discussion in van Nieukerken et al. (2012). As there are no Syntypes to select as Lectotype, we suggest that a reared male specimen should be selected as Neotype, preferably collected as close to the type locality as possible.

##### Material: Adults examined.

CANADA – **Ontario** • 3 ♀; Normandale; 42.71N, 80.31W; Freeman, Lewis leg.; *Parthenocissus*; emerged 15–23 Mar. 1958; EventId: 57–157; Genitalia slide: MIC1865, MIC1869, MIC1867; CNCLEP00100465, 00122296, 00122301. • 1 ♂; Normandale; 42.71N, 80.31W; Freeman, Lewis leg.; *Parthenocissus quinquefolia*; emerged 13 Mar. 1958; EventId: 57–157; Genitalia slide: MIC1866; CNCLEP00100464. • 6; Normandale; 42.71N, 80.31W; Freeman & Lewis leg.; *Parthenocissus* (*quinquefolia)*; emerged 14–19 Mar. 1958; EventId: 57–157; CNCLEP00122294, 00122295, 00122297–00122300. • 1 ♂; Overbrook; 45.42N, 75.65W; G.G. Lewis leg.; *Parthenocissus vitacea*; emerged 05 Jul. 1956; EventId: 55–57; CNCLEP00122409. • 1; Simcoe; 42.83N, 80.31W; T.N. Freeman leg.; emerged 16 Jan. 1960; EventId: 65–80; CNCLEP00100466.

USA – **Connecticut** • 1 ♂; Winham Co, Windham airport, Mansfield Hollow SP; 41.74783N, 72.16409W; alt. 80 m; 09 Sep. 2011; E.J. van Nieukerken leg.; *Parthenocissus quinquefolia*; emerged 24 Apr. 2012; EventId: EvN no 2011178–2K; Genitalia slide: EvN4377; RMNH.INS.24377. • 1; same data as preceding; emerged 05 May. 2012; RMNH. – **Massachusetts** • 1 ♂; Hampshire Co., Pelham, 88 Arnold Rd.; 42.3629N, 72.4598W; 30 Aug. 2013; C.S. Eiseman leg.; *Parthenocissus quinquefolia*; emerged 13 May. 2014; EventId: CSE1108; CSEC. • 1 ♀; Hampshire Co., Pelham, 88 Arnold Rd.; 42.3629N, 72.4598W; 04 Sep. 2013; C.S. Eiseman leg.; *Parthenocissus quinquefolia*; emerged 05 May. 2014; EventId: CSE1102; CSEC. – **New York** • 1 ♂; St Lawrence Co., Oak Point; 44.51499N, 75.748734W; 12 Aug. 1988–17 Aug. 1988; D.L. Wagner leg.; *Parthenocissus quinquefolia*; emerged 14 Mar. 1989; Genitalia slide: EvN4200; RMNH.INS.24200; Wagner, D.L., personal collection. – **Oklahoma** • 1; Payne Co., Mehan; 36.014339N, 96.996744W; 26 Jun. 2016; M.W. Palmer leg.; *Parthenocissus quinquefolia*; emerged 16 Apr. 2017; EventId: CSE3567; CSEC. – **Vermont** • 3 ♂; Addison Co, Button Bay SP, Lake Champlain borders; 44.18154N, 73.36892W; alt. 40–50 m; 16 Sep. 2011; E.J. van Nieukerken leg.; *Parthenocissus quinquefolia*; emerged 23 Apr. 2012; EventId: EvN no 2011254–1K; Genitalia slide: EvN4376; RMNH.INS.24376. • 1 ♀; Salisbury Co., Bryant Mtn., 16 Pudding Hill Rd.; 44.54347N, 72.009928W; 10–11 Sep. 1987; D.L. Wagner leg.; *Parthenocissus quinquefolia*; emerged 29 Apr. 1988; Genitalia slide: JCK15220; Wagner, D.L., personal collection.

##### Larvae and leafmines examined.

USA – **Massachusetts** • Franklin Co., Northfield; 42.646558N, 72.426541W; 16 Sep. 2016; Charley Eiseman leg.; *Parthenocissus quinquefolia*; CSEC. • Hampshire Co., Pelham; 42.363212N, 72.460107W; 30 Aug. 2013; Charley Eiseman leg.; *Parthenocissus quinquefolia*; EventId: CSE914; CSEC; • 1 larva; Nantucket Co., Nantucket, Squam Swamp; 41.319937N, 70.00244W; alt. 2 m; 07 Sep. 2011; C.S. Eiseman leg.; *Parthenocissus quinquefolia*; EventId: C4; RMNH.INS.29941. • Plymouth Co., Bridgewater; 41.985542N, 71.044771W; 16 Aug. 2013; C.S. Eiseman leg.; *Parthenocissus*; EventId: CSE983; RMNH. – **Vermont** • Chittenden Co., Williston, Mud Pond; 44.413625N, 73.075697W; 28 Aug. 2016; Charley Eiseman leg.; *Parthenocissus quinquefolia*; EventId: CSE3764; CSEC.

##### BOLD data, material not examined.

CANADA – **Ontario** • 1; St Williams, Turkey Point Provincial Park; 42.7052N, 80.3285W; alt. 222 m; 23 Jun. 2014–07 Jul. 2014; CBG Collections Staff leg.; EventId: GMP#03287; BIOUG35664–A01. • 1; Thorold, Short Hills Provincial Park; 43.1129N, 79.2738W; alt. 94 m; 23 Jun. 2014–07 Jul. 2014; CBG Collections Staff leg.; EventId: GMP#03255; BIOUG35571–A12.

##### Observations.

USA – **Massachusetts** • Nantucket Co., Nantucket, Stump Pond; 41.292454N, 70.002666W; 05 Sep. 2019; Charley Eiseman leg.; *Parthenocissus quinquefolia*; https://www.inaturalist.org/observations/44821470. • **New York** • Putnam Co., Putnam Valley, 392 Dennytown Rd.; 41.41309N, 73.86508W; 09 Aug. 2016; Even Dankowicz leg.; *Parthenocissus*; https://www.inaturalist.org/observations/17908231, https://www.inaturalist.org/observations/17908234. – **Rhode Island** • Washington Co., Block Island; 41.195N, 71.5652W; 08 Aug. 2019; Aaron Hunt leg.; *Parthenocissus*; https://bugguide.net/node/view/1741669/bgimage. – **Vermont** • Chittenden Co., Shelburne; 44.39445N, 73.23003W; 14 Sep. 2019; Spencer Hardy leg.; *Parthenocissus quinquefolia*; https://www.inaturalist.org/observations/32714140. – **Wisconsin** • Dane Co., Madison, UW Lakeshore Nature Preserve; 43.084N, 89.429W; 25 Aug. 2019; Tom Klein leg.; *Parthenocissus quinquefolia*; https://bugguide.net/node/view/1717010/bgimage.

#### Aspilanta voraginella

Taxon classificationAnimaliaLepidopteraHeliozelidae

(Braun, 1927), comb n.

123275DC-3FC2-5F91-978E-87718A5FC0C7

Figs 5 , 12 , 24 , 33 , 80–83

Antispila voraginella Braun, 1927b: 191. Holotype ♂: USA: [Utah: Washington County] “B1206/Zion Canyon/Utah i.iv.9 [1926]- Antispila / voraginella / Type Braun.”, Genitalia slide EJvN 3916 [reared from mines on Vitis arizonica] (ANSP) [examined].
Antispila voraginella ; Needham et al. 1928: 290; McDunnough 1939: 91; Davis 1983b: 4; Powell and Opler 2009: 39; van Nieukerken et al. 2012: 54; Eiseman 2019: 732.

##### Differential diagnosis.

Wingspan ca. 4.7–5.4 mm, forewing length 2.3–2.6 mm. Externally separated from *A. oinophylla* and *ampelopsifoliella* by the darker brassy brown head scaling, the absence of a fringe line and the overall less shining metallic pattern. Relatively similar to *A. argentifera*, but unlikely to be sympatric with that species. Male genitalia with distinctly bilobed tegumen, fewer pecten sensilla (8–10) and phallotrema with smaller spines only.

**Figures 34–42.**
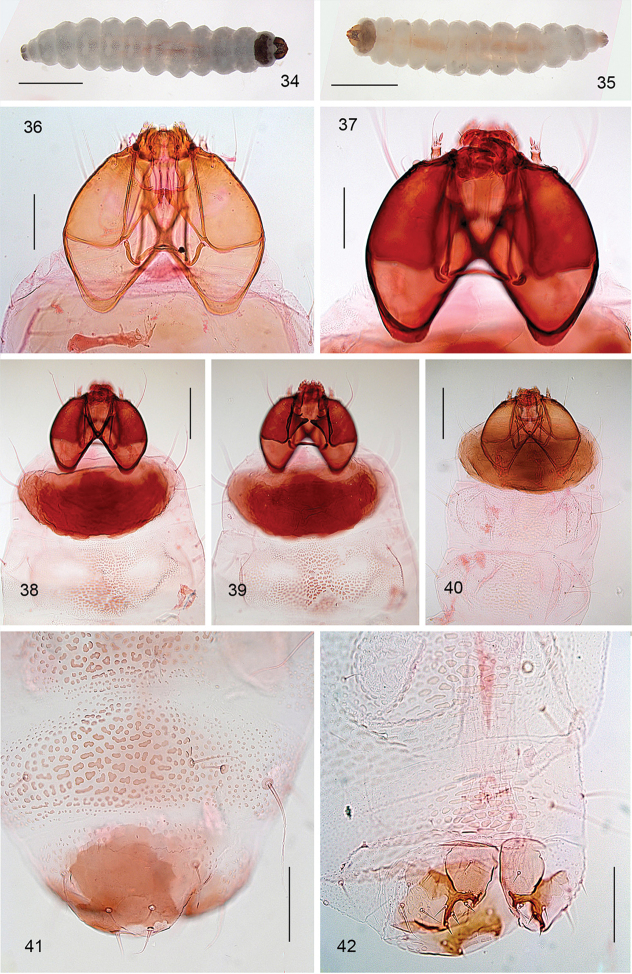
*Aspilanta* species, larval morphology, final feeding instar (4^th^) and non-feeding instar (5^th^, **36**). **34***A. ampelopsifoliella*, ventral aspect, ethanol preserved larva, RMNH.INS.18672P **35***A. viticordifoliella*, ventral aspect, ethanol preserved larva, RMNH.INS.18509P **36***A. argentifera*, head capsule, RMNH.INS.18566P **37–39***A. ampelopsifoliella*, head and thorax, resp. focussed at mid levels (head only), dorsally and ventrally, RMNH.INS.18672P **40***A. oinophylla*, head and thorax, RMNH.INS.18394P **41***A. ampelopsifoliella*, last 3 abdominal segments, dorsal aspect, RMNH.INS.18672P **42***A. oinophylla*, last 3 abdominal segments, ventral aspect, RMNH.INS. 18394P. Scale bars: 1 mm (**34, 35**), 100 μm (**36, 37, 41, 42**), 200 μm (**38–40**).

**Figure 43.**
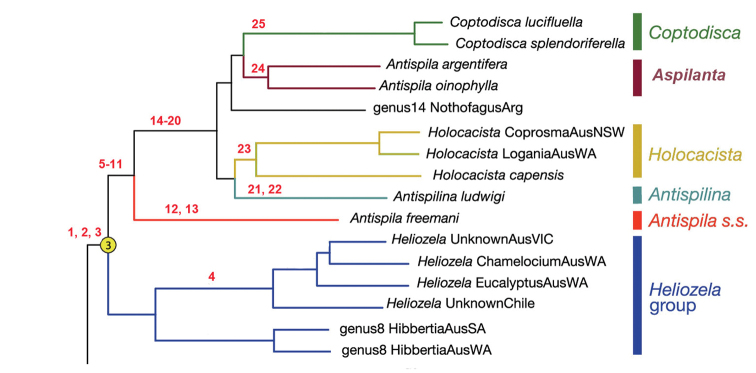
Phylogeny of the cosmopolitan leafmining clade of Heliozelidae, part of fig. 1 in Milla et al. (2019) (*Maximum likelihood phylogeny generated using iq-tree, topology from filtered_nt123 analysis*). ‘*Antispila*’ Group II replaced by *Aspilanta*, branch supports removed and numbers of possible apomorphies added; see text.

**Figure 44.**
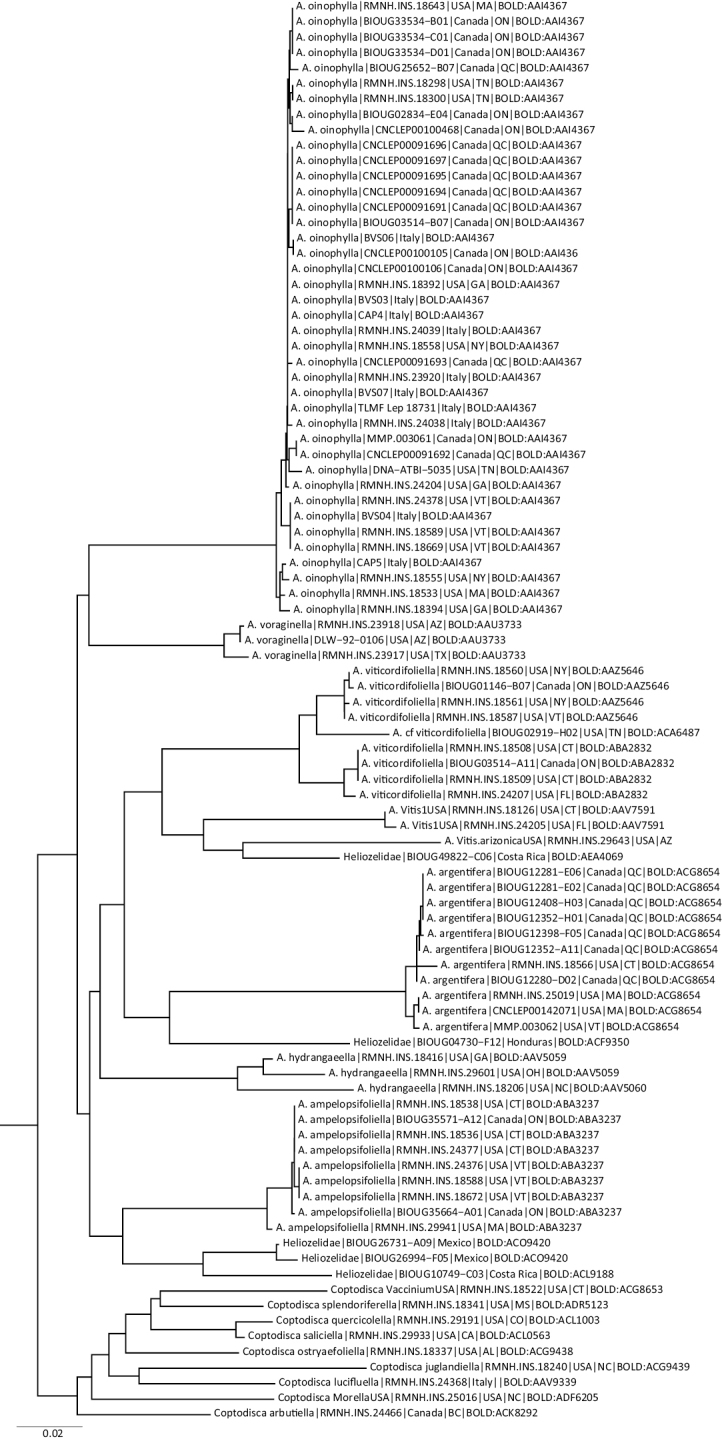
Neighbor-Joining tree of COI barcodes of *Aspilanta* species, including Nearest Neighbours. Several species of *Coptodisca* serve as outgroup. Data with country, and for Canada and United States also the abbreviation for Province or State, plus the BIN number.

##### Host plant.

Vitaceae: *Vitis arizonica*, unidentified *Vitis*.

##### Leafmines.

(Figs 80–83) Yellowish white blotches, without any linear part; greenish black frass irregularly in centre; mine usually formed from the confluence of several mines; as many as twenty or twenty-five pupal cases may be cut from a single leaf.

##### Larva.

Pale yellowish, head and prothorax hardly darker, mouthparts stand out as darker brown; a row of 5–7 brown spots on abdomen visible (Fig. 80). Larvae gregarious.

##### Life history.

Larvae found between 4 June and 9 August (Arizona); in all cases the adults emerged the next spring (April to June), suggesting that the species is univoltine.

##### Distribution.

USA: Arizona, Texas (west), Utah.

##### Barcode.

BIN: BOLD:AAU3733, average distance 0.92%, max. distance 1.38% (*n* = 3), distance to nearest neighbour 8.83% (BOLD: AAV5059, *A. hydrangaeella*).

##### Parasitoids.

None known.

##### Material.

Adults: see van Nieukerken et al. (2012).

##### Observations.

USA – **Arizona** • Cochise Co., Portal; 31.90918N, 109.25228W; 03 Aug. 2019; Laura Gaudette leg.; *Vitis*; https://www.inaturalist.org/observations/30135988, https://www.inaturalist.org/observations/32296900. – **Utah** • Washington Co., Zion National Park; 37.23N, 112.963W; 22 Jul. 2008; Charley Eiseman & Noah Charney leg.; *Vitis*; https://www.inaturalist.org/observations/44822019.

#### Aspilanta argentifera

Taxon classificationAnimaliaLepidopteraHeliozelidae

(Braun, 1927) comb. n.

B8E5A82C-5F49-5C2D-837B-1F6FA292AE7B

Figs 4 , 6 , 13 , 29 , 31 , 36 , 49 , 88–96

Antispila argentifera Braun, 1927a: Holotype ♀, Canada, Ontario, Sparrow Lake, 16.vii.1926, A. Braun, on young leaves of birch. (ANSP) [Photograph examined, Fig. 6.]
Antispila argentifera ; Needham et al. 1928: 289; McDunnough 1939: 91; Davis 1983b: 4; van Nieukerken and Pohl 2018: 42; Milla et al. 2017: 140; Eiseman 2019: 189, 190, 976, 1082, 1085, 1090, 1093.
Antispila species on sweet fern; Forbes 1923: 227.
Antispila n. sp., Grehan et al. 1995: 46 [Vermont, from Comptonia peregrina, det. D.L. Wagner]

##### Differential diagnosis.

Wingspan ca. 4.0–5.2 mm, forewing length 1.8–2.4 mm. Externally separable from *A. oinophylla* and *ampelopsifoliella* by the darker brassy head scaling, the grey fringe and the indistinct fringe line; *A. argentifera* is also smaller. Relatively similar to *A. voraginella* which is only known from the Southwestern states. Male genitalia with tegumen distinctly bilobed, valvae with 9–12 pecten sensilla, phallus without strong appendices (only one slide examined). Mines on *Morella* distinguished from those of an unnamed *Coptodisca* species, which occurs especially in the Southeast, by the larger cut-out, and the overall larger mine.

**Figures 45–50.**
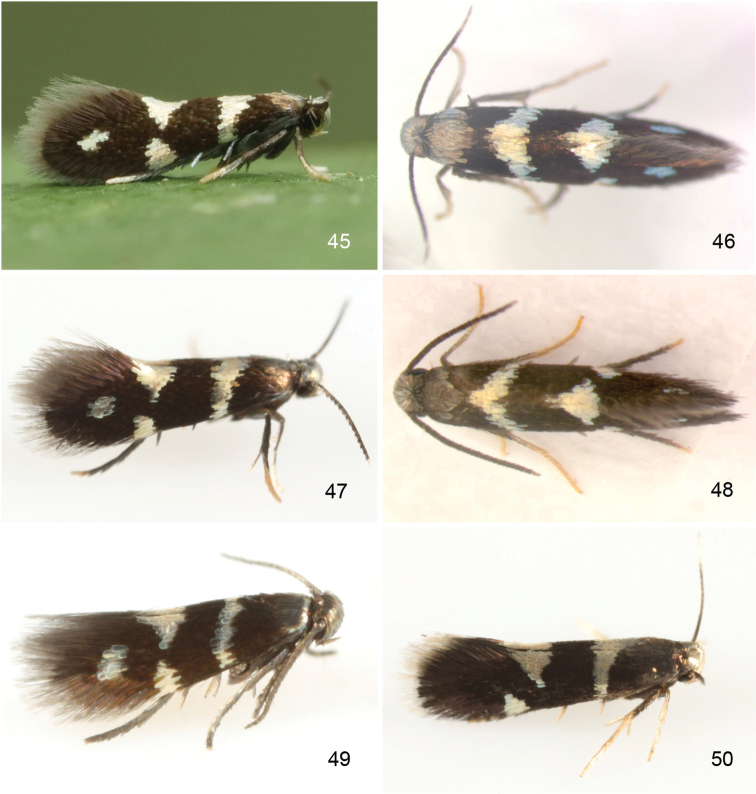
*Aspilanta* species, live adult moths. **45***A. oinophylla*, male, Italy, Südtirol, Eisacktal, Klausen, 650 m, e.l. 1.viii.2019, photograph Dieter Robrecht **46***A. hydrangaeella*, female, dorsal view, NC, photograph EJvN **47***A. ampelopsifoliella*, female, Pelham MA, photograph CSE **48***A. ampelopsifoliella*, male, dorsal view, VT, photograph EJvN **49***A. argentifera*, female, Stockbridge MA, photograph CSE **50***A. viticordifoliella*, female, Pelham MA, photograph CSE.

**Figures 51–58.**
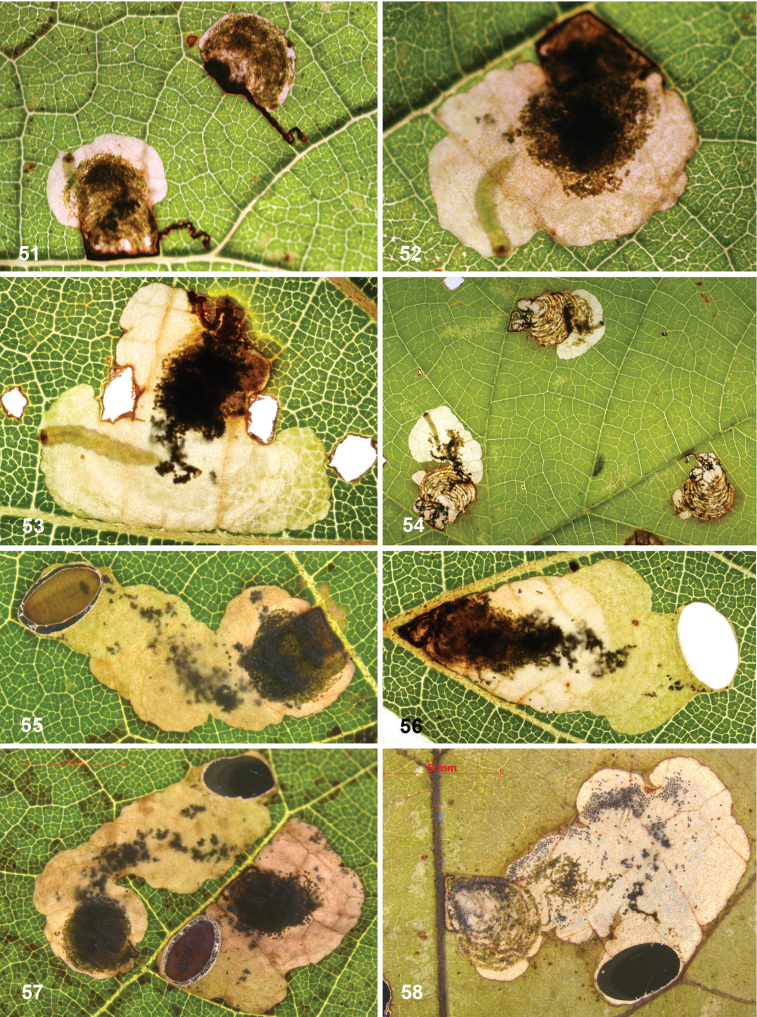
*Aspilanta oinophylla*, leafmines, larvae and shields. **51, 52** GA, Chattahoochee NF, 14.x.2010, type locality, *Vitis aestivalis*, EvN2010266 **53, 56 MA**, Northfield, 13.ix.2016, *V. riparia*, CSE **54, 58** TN, Great Smoky Mts NP, 2.x.2010, *V. vulpina*, EvN2010119 **55, 57** VT, Button Bay SP, 16.ix.2011, *V. riparia*, EvN2011253.

##### Host plants.

Myricaceae: *Comptonia peregrina*, *Morella caroliniensis* (including *M. pensylvanica*, cf. Wilbur (2002)), *M. cerifera*, *Myrica gale*.

##### Leafmines.

(Figs 88–96) Egg in the examined mines inserted at the midrib. The mine starts with a linear portion, following the midrib towards the apex, sometimes difficult to see when it is very close to the midrib (Fig. 94); later becoming a small blotch extending from midrib to leaf margin. Frass blackish, varying from dispersed to concentrated near origin of mine; in linear portion a wide line in middle. Mines on *Comptonia* are usually very compact, on *Morella* and *Myrica* more elongate; on *Myrica gale* we observed several mines crossing the midrib, resulting in a wilting leaf tip that may drop off (Fig. 90). The larva cuts out an elliptic case ca. 2.5–3.5 mm long.

##### Larva.

Pale yellowish green, head and prothorax brown.

##### Life history.

Bivoltine. Larvae found in July and September to November, adults in June, July and August; specimens reared from fall mines emerged (indoors) in April to May, from July mines in August. Mines of the first generation seem to be very scarce.

##### Distribution.

Canada: Newfoundland*, Nova Scotia, Ontario, Prince Edward Island*, Quebec*; USA: Connecticut*, Massachusetts*, New York*, North Carolina*, Vermont.

##### Barcode.

BIN: BOLD:ACG8654, average distance 0.66%, max. distance 2.25% (*n* = 15), distance to nearest neighbour 11.67% (BOLD:ACF9350, an unidentified Heliozelidae from Honduras).

##### Parasitoids.

Eulophidae: *Pediobius ? albipes* (Provancher, 1887) (NY), *Pnigalio* sp. (MA).

##### Remarks.

Annette Braun (1927a) believed that the moth that she collected at Sparrow Lake belonged to long leafmines that she found on *Betula* in the same spot. Her description of the mine actually matches very well that of *Phylloporia bistrigella* (Haworth, 1828) (Incurvariidae) (Eiseman 2019; Ellis 2020), a Holarctic species, widespread in Canada, but in 1927 poorly known in North America as either *Incurvaria labradorella* Clemens, 1864 or *I. aureovirens* Dietz, 1905, which had not yet been associated with *Betula* and which were only later synonymised with *P. bistrigella* by Davis (1983a). On the basis of Braun’s record of these leafmines, *P. bistrigella* should also be listed for Ontario, a record missing from Pohl (2018). As we have never seen any heliozelid mine on *Betula*, and as the Holotype of *A. argentifera* externally matches the specimens reared from Myricaceae, we are convinced that *argentifera* is the correct name for this species (see also Milla et al. 2017: 140). According to Braun (1927a), the holotype was a male, but the photograph (Fig. 6), kindly made by Mike Palmer, clearly shows a female, with protruding oviscapt. We refrain for now from borrowing and dissecting the holotype, as the characters of female genitalia have not been sufficiently studied, and a future dissection could possibly be combined with newer techniques for obtaining DNA data. Already Forbes (1923) reported an *Antispila* species from sweet fern (= *Comptonia*), which strange enough according to him “has not been distinguished from *A. isabella*”.

Specimens from Prince Edward Island were previously regarded as misidentifications (van Nieukerken and Pohl 2018) but are here re-identified as correct *A. argentifera*.

##### Material: Adults examined.

CANADA – **Nova Scotia** • 1 ♂; Barrington; 43.56N, 65.57W; G.G. Lewis leg.; *Comptonia peregrina*; emerged 20 Aug. 1970; EventId: 70–28; CNCLEP00122407. – **Ontario** • 1 ♀ (Holotype, photograph examined, Fig. 6); Sparrow Lake; 44.81N, 79.4W; 16 Jul. 1926; A.F. Braun leg.; ANSP. – **Prince Edward Island** • 1 ♂; Dalvay House, Can. Nat. Park; 46.416N, 63.073W; 19 Jul. 1940; G.S.Walley leg.; Genitalia slide: MIC7517; CNCLEP00100470. • 1 ♂; same data as preceding; Genitalia slide: T.N.F. No. 405; CNCLEP00100469.

USA – **Massachusetts** • 1 ♀; Berkshire Co, Stockbridge, Kampoosa Bog; 42.294364N, 73.304347W; 16 Jul. 2017; C. S. Eiseman leg.; *Myrica gale*; emerged 09 Aug. 2017; EventId: CSE4096; RMNH. • 1 ♀; Franklin Co., Erving; 42.618565N, 72.422256W; 27 Oct. 2014; C. S. Eiseman leg.; *Comptonia peregrina*; emerged 18 May. 2015; EventId: CSE1557; Genitalia slide: EvN5019; RMNH.INS.25019. • 1 ♂; Franklin Co., Erving; 42.618565N, 72.422256W; 27 Oct. 2014; C. S. Eiseman leg.; *Comptonia peregrina*; emerged 18 May. 2015; EventId: CSE1557; CNCLEP00142071. • 1 ♀; Nantucket Co., Nantucket, Radar Hill; 41.28390847N, 70.03875002W; 15 Nov. 2015; C. S. Eiseman leg.; *Morella caroliniensis*; emerged 18 Apr. 2016; EventId: CSE2398; RMNH. • 1 ♂ 1 ♀ (in copula); Nantucket Co., Nantucket, State Forest; 41.26059N, 70.07991W; alt. 9 m; 10 Jun. 2013; C. Eiseman leg.; EventId: CSE564; Genitalia slide: EvN5193; RMNH.INS.25193. • 1; Nantucket Co., Nantucket, State Forest; 41.26059N, 70.07991W; alt. 9 m; 10 Jun. 2013; C. Eiseman leg.; EventId: CSE563; RMNH. • 1 ♀; Nantucket Co., UMass field station; 41.2942N, 70.0399W; 05 Nov. 2017; C.S. Eiseman leg.; *Morella caroliniensis*; emerged 18 May. 2018; EventId: CSE4546; CSEC. – **New York** • 3; Orange Co., Cornwall, Black Rock Forest; 41.396209N, 74.025219W; 30 Aug. 2019; Charley Eiseman & Julia Blyth leg.; *Comptonia peregrina*; emerged 07–30 Apr. 2020; EventId: CSE6145, CSE6169, CSE6188; CSEC.

##### Larvae and leafmines examined.

USA – **Connecticut** • 1 larva, leafmines; New London Co, Connecticut College Arboretum; 41.37929N, 72.11121W; alt. 60 m; 10 Sep. 2011; E.J. van Nieukerken leg.; *Morella caroliniensis*; EventId: EvN no 2011194–2M; RMNH.INS.18566.P, RMNH.INS.43558, RMNH.INS.43559. – **Massachusetts** • vacated mines; Berkshire Co., Beartown State forest, Benedict Pond; 42.20288N, 73.28913W; alt. 485 m; 12 Sep. 2011; E.J. van Nieukerken leg.; *Comptonia peregrina*; EventId: EvN no 2011211–H; RMNH.INS.43586. • Franklin Co., Northfield; 42.646851N, 72.4247W; 26 Oct. 2015; Charley Eiseman leg.; *Comptonia peregrina*; CSEC. • Franklin Co., Erving; 42.618565N, 72.422256W; 27 Oct. 2014; Charley Eiseman & Julia Blyth leg.; *Comptonia peregrina*; EventId: CSE1479; CSEC. • Nantucket Co., Nantucket, Pout Ponds; 41.277N, 70.046W; 03 Nov. 2013; Charley Eiseman & Julia Blyth leg.; *Morella caroliniensis*; CSEC. • Nantucket Co., Nantucket, UMass field station; 41.295464N, 70.039325W; 05 Nov. 2017; Charley Eiseman & Julia Blyth leg.; *Morella caroliniensis*; EventId: CSE4445; CSEC. – **New York** • vacated mines; Essex Co, S Wilmington, W branch Ausable river; 44.3662N, 73.84118W; alt. 340 m; 13 Sep. 2011; E.J. van Nieukerken leg.; *Comptonia peregrina*; EventId: EvN no 2011223–3H; RMNH.INS.43610. – **Vermont** • 3 larvae (used for transcriptome studies), leafmines; Washington Co., North Montpelier, Chickering Bog; 44.3247N, 72.48089W; alt. 365 m; 06 Sep. 2015; E.J. van Nieukerken, C. Eiseman & J. Blyth leg.; *Myrica gale*; EventId: EvN no 2015222–2M; RMNH.INS.30599, 30600, 30601, RMNH.INS.40367, RMNH.INS.40631.

##### BOLD data, material not examined.

CANADA – **Newfoundland and Labrador** • 1 ♀; Barachois Pond Provincial Park, Erin Mtn trail; 48.469N, 58.256W; alt. 240 m; 29 Jun. 2011; G.R.Pohl, D.W.Langor leg.; CCDB–23267–F01; NFC. • 1 ♂; J. T. Cheeseman Provincial Park; 47.633N, 59.255W; 27 Jun. 2011; G.R.Pohl, L.Lafosse leg.; CCDB–23267–E06; NFC. • 1 ♀; Hwy 1, 5 km N jct Rte 480; 48.561N, 58.264W; alt. 138 m; 25 Jun. 2011; G.R.Pohl, D.W.Langor leg.; CCDB–23267–F05; NFC. – **Quebec** • 3; Mingan Archipelago National Park Reserve, Quarry Island; 50.2135N, 63.7979W; 19–26 Jun. 2013; Park Staff leg.; EventId: GMP#01092; BIOUG12280–D02, BIOUG12281–E02, BIOUG12281–E06. • 3; same locality data as preceding; 02–09 Jul. 2013; Park Staff leg.; EventId: GMP#01096; BIOUG12352–A11, BIOUG12352–H01, BIOUG12398–F05. • 1; same locality data as preceding; 16–24 Jul. 2013; Park Staff leg.; EventId: GMP#01100; BIOUG12408–H03.

##### Observations.

USA – **Massachusetts** • Plymouth Co., Marshfield, Hoyt Hall Preserve; 42.066N, 70.678W; 29 Oct. 2016; Steven Whitebread leg.; *Morella caroliniensis*; (photographs examined). – **New York** • Orange Co., Cornwall, Black Rock Forest; 41.39621N, 74.02522W; 30 Aug. 2019; Charley Eiseman & Julia Blyth leg.; *Comptonia peregrina*; https://www.inaturalist.org/observations/37506602. – **North Carolina** • Scotland Co., Laurinburg, St. Andrews University; 34.747N, 79.477W; 27 Sep. 2016; Tracy Feldman leg.; *Morella cerifera*; https://bugguide.net/node/view/1298044/bgimage same locality as preceding; 08–09 Nov. 2016; Tracy Feldman leg.; *Morella cerifera*; https://bugguide.net/node/view/1312510/bgimage, https://bugguide.net/node/view/1312770/bgimage • same locality as preceding; 16 Oct. 2017; Tracy Feldman leg.; *Morella cerifera*; https://bugguide.net/node/view/1456019/bgimage.

#### Aspilanta viticordifoliella

Taxon classificationAnimaliaLepidopteraHeliozelidae

(Clemens, 1860), comb n.

909BD0C7-3CF4-5ADD-9BED-54985131E520

Figs 8 , 35 , 50 , 97–106

Antispila viticordifoliella Clemens, 1860: 209. Syntype mines, larva [USA: Pennsylvania, Easton], larvae on “wild grapes” [Vitis vulpina], August–September, Brackenridge Clemens (ANSP if extant).
Antispila viticordifoliella ; Chambers 1874a: 168; Frey and Boll 1878: 253; Chambers 1880: 65; Edwards 1889: 125; Dyar et al. 1903: 539; Barnes and McDunnough 1917: 181; Forbes 1923: 226; Needham et al. 1928: 20, 153, 290; McDunnough 1939: 91; Davis 1983b: 4; Brower 1984: 29; McGiffen and Neunzig 1985: 55; van Nieukerken and Pohl 2018: 41; Eiseman 2019: 189, 190, 729, 733.
Antispila cf. viticordifoliella van Nieukerken et al. 2012: 58; Eiseman 2019: 729.

##### Differential diagnosis.

Wingspan ca. 5.5 mm, forewing length 2.2–2.5 mm. Externally different from all other *Aspilanta* species by the absence of the apical spot in the forewings. Differs from most *Antispila* species, which have a similar wing pattern, by the antennae with distinct white tips, the complete absence of androconial scales in the males and the smaller size. Genitalia not yet examined. Leafmines differ from other *Aspilanta* on Vitaceae by the absence of a linear portion and by the larva that is yellow-green (vs. whitish/colourless in *A. ampelopsifoliella* and *oinophylla*); from *Antispila* species by the black dispersed frass rather than brown and the markedly smaller cut-out. The record of an *Antispila* sp. from similar mines (see Remarks) complicates this, though.

**Figures 59–68.**
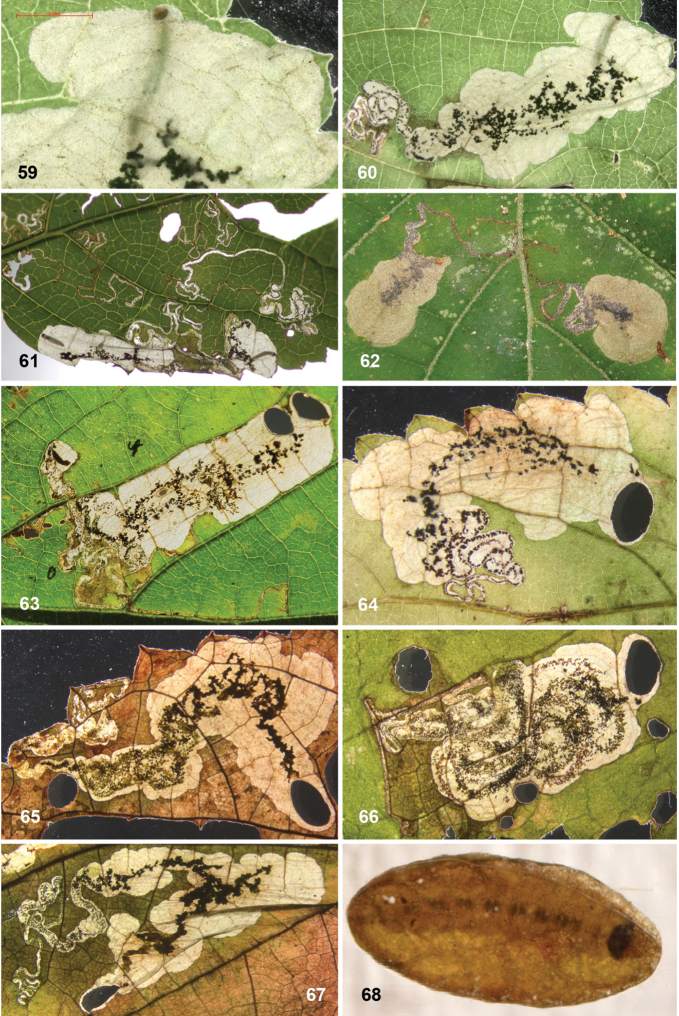
*Aspilanta hydrangaeella*, leafmines, larvae and shields, on *Hydrangea arborescens*. **59, 60, 64** GA, Chattahoochee NF, 14.x.2010, EvN2010279 **61, 63, 68** OH, South Bloomingville, 13.ix.2012, CSE, (61=RMNH.INS.29601) **62** OH, Crane Hollow, 5.viii.2016, CSE **65–67** NC, Great Smoky Mts NP, 28.ix.2010, EvN2010073.

**Figures 69–79.**
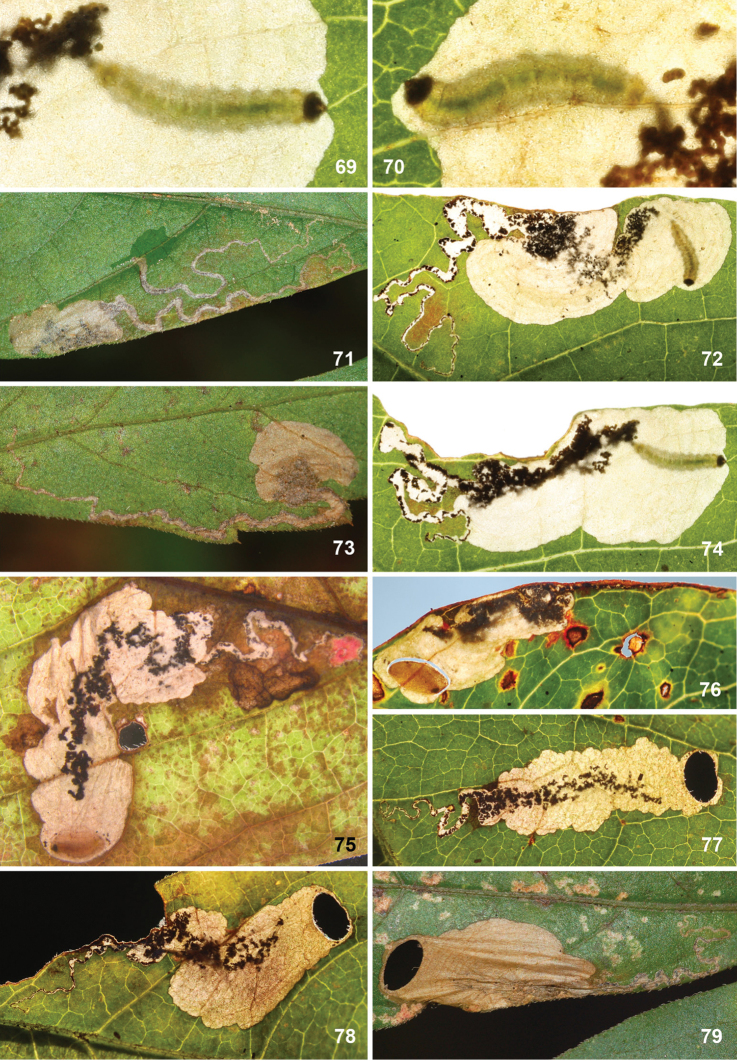
*Aspilanta ampelopsifoliella*, leafmines, larvae and shields, on *Parthenocissus quinquefolia*. **69, 74** VT, Williston, 28.viii.2016, CSE3764 (same mine) **70, 76–78** MA, Pelham, 30.viii, 4.ix.2013, CSE1102, 1108 **71** MA, Nantucket, Squam Swamp, 7.ix.2011, CSE-C4 **72** MA, Northfield, 16.ix.2016, CSE **73** MA, Nantucket, Stump Pond, 5.ix.2019, CSE **75** VT, Button Bay SP, 16.ix.2011, EvN2011254 **79** MA, Bridgewater, 16.viii.2013, CSE983.

##### Host plants.

Vitaceae: *Parthenocissus quinquefolia*, *P. vitacea, Vitis vulpina*.

##### Leafmines.

(Figs 97–106) Egg in the majority of examined mines in leaf margin, occasionally anywhere in the leaf, usually not associated with veins. The mine starts almost immediately as a blotch, occasionally with a minute linear portion along leaf margin; blotch rather compact, often along leaf margin or slightly extending towards middle, in narrow leaflets until midrib. Frass black, a thick clump near origin, more dispersed in centre of mine. The larva cuts out an elliptic case ca. 3.5–4 mm long.

##### Larva.

Yellow-green except for green gut contents, in contrast with colourless or whitish larva of *A. ampelopsifoliella* on the same host plant (compare Figs 69, 70 with 97–100); head and prothorax dark brown.

##### Life history.

Larvae found from August to September through most of the range, but in Florida in April. Field caught adults found in July; larvae collected in fall resulted in emerging adults in April-May, the adult from the April larva emerged in May.

##### Distribution.

Canada: Ontario, Québec*; USA: Connecticut, Florida, Kentucky, ? Maine, Massachusetts*, New York, Pennsylvania, ? Texas, Vermont. The previous record from Vermont by Grehan et al. (1995) was incorrect, as the cited material in the collection of D.L. Wagner belongs to an *Antispila* species. Records from Maine (Brower 1984) and Texas (Frey and Boll 1878) are uncertain; the last authors give a description but did not mention the white-tipped antenna.

##### Barcode.

BIN: BOLD:ABA2832 (CT, FL), average distance 0.32%, max. distance 0.8% (*n* = 5), distance to NN 3.53% (next BIN), BIN: BOLD:AAZ5646 (NY, VT), average distance 0.25%, max. distance 0.46% (*n* = 4), distance to NN 2.89% (BOLD:ACA6487). The single specimen in a Malaise trap with that BIN: BOLD:ACA6487, most likely also belongs to *A. viticordifoliella*, to be confirmed by morphological examination.

##### Remarks.

*Antispila viticordifoliella* was described on the basis of larvae and leafmines on *Vitis vulpina* (as *V. cordifolia*), with insufficient detail to recognise the species. The first adults were described by Chambers (1874a), again as new species. He clearly described a moth, reared from the same host, without an apical spot and antennae “with about six terminal joints silvery white”. His description has later served as basis for the identity of this species, and one of the specimens in MCZ from his collection, incorrectly termed “types” might probably be the best to serve as Neotype, after a male has been dissected and DNA been extracted. Unfortunately the specimens are rather worn, and antennae are missing. The name has also several times been incorrectly used for one of the true *Antispila* species on *Vitis*, *A. isabella* Clemens, 1860 or a related species (see van Nieukerken et al. 2012).

Earlier we excluded the specimens reared from *Parthenocissus* from this species (van Nieukerken et al. 2012), but we are now more convinced that they are conspecific. Since then it has become clear that Vitaceae-feeding Heliozelidae frequently use more than one plant genus as host (eg van Nieukerken and Geertsema 2015), as we also observe in *A. oinophylla*. Still we urge that a species level revision using more material should be carried out.

From very similar mines in *Parthenocissus* collected in North Carolina, CSE and Tracy Feldman have reared an unidentified species of *Antispila*; we have thus omitted possible records of *Aspilanta viticordifoliella* from Iowa, Kansas and Ohio that are based only on photographed mines. In photographs of the North Carolina mines taken in the field, the larvae appear to be paler than those of *A. viticordifoliella*, but this requires confirmation; the *Antispila* mines are otherwise only recognisable when the typical keeled case is formed. The reared adults in fact also resemble *A. viticordifoliella* because of the white-tipped antennae; only checking wing venation and/or the genitalia will separate the two.

##### Material: Adults examined.

CANADA – **Ontario** • 1 ♀; Ottawa; 45.41N, 75.69W; G.G. Lewis leg.; emerged 02 Apr. 1971; EventId: 70–48; Genitalia slide: MIC1876; CNCLEP00100475.

USA – **Massachusetts** • 1 ♀; Hampshire Co., Pelham, 88 Arnold Rd.; 42.3629N, 72.4598W; 30 Aug. 2013; C.S. Eiseman leg.; *Parthenocissus quinquefolia*; emerged 13 May. 2014; EventId: CSE1109; CSEC.

##### Larvae and leafmines examined.

CANADA – **Québec** • 1 vacated mine; Gatineau, Aylmer E, near Ottawa river; 45.39023N, 75.79139W; alt. 56 m; 23 Aug. 2015; E.J. van Nieukerken leg.; *Parthenocissus vitacea*; EventId: EvN no 2015160–1H; RMNH.INS.40247.

USA – **Massachusetts** • Hampshire, Northampton Co., Northampton Bikeway west of King St.; 42.329151N, 72.637529W; 13 Sep. 2013; Charley Eiseman leg.; *Parthenocissus quinquefolia*; CSEC. – **Vermont** • Chittenden Co., Williston, Mud Pond; 44.413625N, 73.075697W; 28 Aug. 2016; Charley Eiseman leg.; *Parthenocissus quinquefolia*; CSEC.

##### BOLD data, material not examined.

CANADA – **Ontario** • 1; Point Pelee National Park, Cactus Field; 41.939N, 82.516W; alt. 168 m; 27 Jun.–04 Jul. 2012; Tyler Peters leg.; EventId: GMP#00175; BIOUG03514–A11. • 1; Wellington County, Puslinch Township, Concession 11/Hume Rd; 43.537N, 80.134W; alt. 320 m; 18–25 Jul. 2010; Paul Hebert leg.; EventId: L#PHPUS–017; BIOUG01146–B07.

USA – **Tennessee** • 1 ♀; Great Smoky Mountains National Park, Twin Creeks Science and Education Center; 35.6859N, 83.4986W; alt. 559 m; 10–17 Jul. 2012; Becky Nichols leg.; EventId: GMP#00037; BIOUG02919–H02.

##### Observations.

CANADA – **Ontario** • Renfrew, Killaloe, Hagarty and Richards; 45.60491N, 77.59134W; 05 Sep. 2019; Jason J. Dombroskie leg.; *Parthenocissus*; https://www.inaturalist.org/observations/32173691. • Renfrew, Wilno; 45.60508N, 77.59131W; 05 Sep. 2019; Jason J. Dombroskie leg.; *Parthenocissus*; https://www.inaturalist.org/observations/32173324.

USA – **Massachusetts** • Berkshire Co., Great Barrington; 42.197001N, 73.335001W; 16 Sep. 2017; Charley Eiseman & Julia Blyth leg.; *Parthenocissus quinquefolia*; https://www.inaturalist.org/observations/44823345.

#### Aspilanta

Taxon classificationAnimaliaLepidopteraHeliozelidae

“Vitis1_USA”

C77E53AB-73CD-599E-8274-FC212C8EF4D6

Antispila Vitis1; van Nieukerken et al. 2012: 56, 77.

##### Differential diagnosis.

Externally not distinguishable from *A. oinophylla* or *A. ampelopsifoliella*. Genitalia and leafmines not yet examined.

##### Host plants.

Vitaceae: *Vitis aestivalis*, *Vitis* spec.

##### Leafmine.

Not examined.

##### Life history.

Larvae in June and July, single adult reared in July. Possibly bivoltine.

##### Distribution.

USA: Connecticut, Florida.

##### Barcode.

BIN: BOLD:ABA2832, average/max. distance 0.35% (*n* = 2), distance to NN 9.72% (BIN: BOLD:AEA4069, unknown, not a public record).

##### Material.

See van Nieukerken et al. (2012).

**Figures 80–87.**
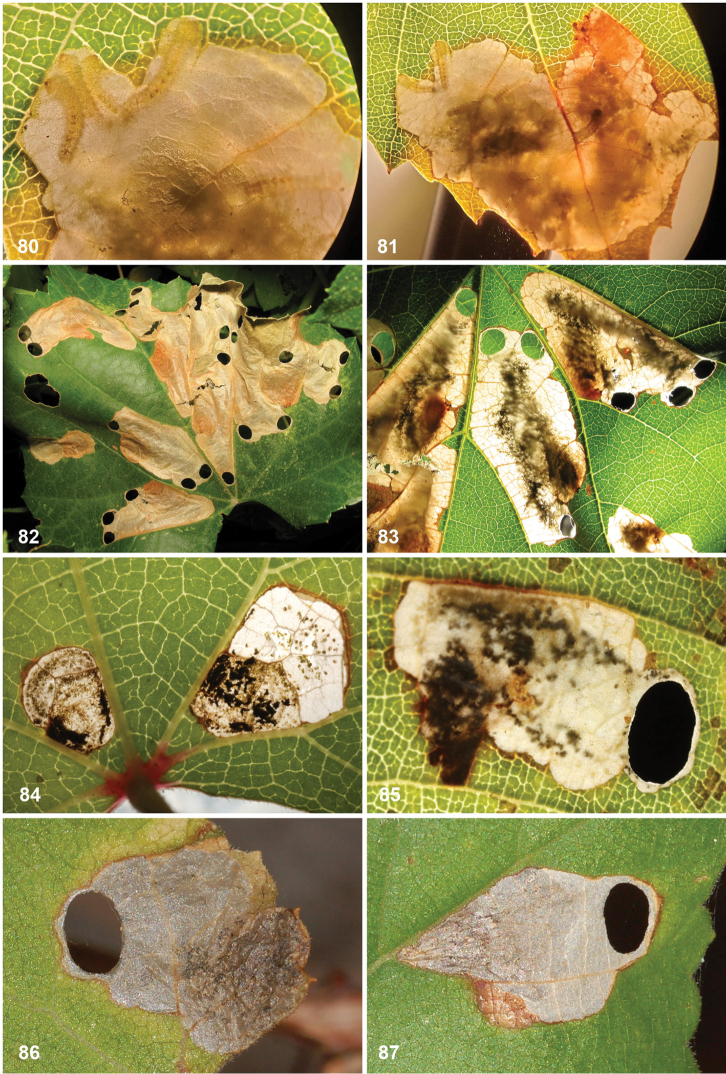
*Aspilanta* from SW USA, leafmines, larvae and shields on *Vitis arizonica*. **80, 81***A. voraginella*, AZ, Cochise Co., 3.viii.2019, photographs by Laura Gaudette **82, 83***A. voraginella*, US, Zion National Park (type locality), 22.vii.2008, lighting from resp,. above and beneath mines, photographs by Noah Charney **84–87***Aspilanta* “Vitis.arizonica_USA”, AZ, Cochise Co., 11.xi.2012, CSE–L141.

**Figures 88–96.**
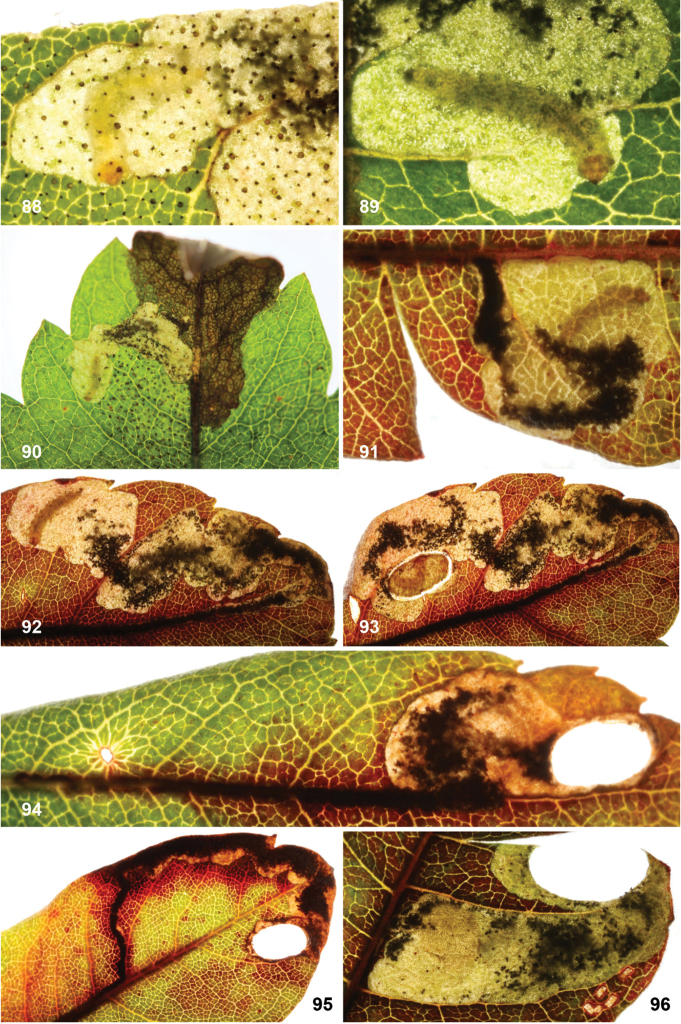
*Aspilanta argentifera*, leafmines, larvae and shields. **88** NC, Laurinburg, 27.ix.2016, *Morella cerifera*, CSE **89** MA, Northfield, 26.x.2015, *Comptonia peregrina*, CSE **90** VT, Chickering Bog, 6.ix.2015, *Myrica gale*, EvN2015222, showing wilted leaf tip **91, 96** MA, Erving, 27.x.2014, *Comptonia peregrina*, CSE1557 **92, 93** MA, Nantucket, Radar Hill, 15.xi.2015, *Morella caroliniensis*, CSE2398, same mine at different stages **94** MA, Stockbridge, 16.vii.2017, *Myrica gale* CSE4096, showing very long linear portion directly along midrib **95** MA, Nantucket, UMass field station, 5.xi.2017, *Morella caroliniensis*, CSE4546.

#### Aspilanta

Taxon classificationAnimaliaLepidopteraHeliozelidae

“Vitis.arizonica_USA”

525B110F-DB7D-5607-8E4B-1F3E701E2D52

Figs 84–87

Antispila sp.; Eiseman 2019: 733.

##### Differential diagnosis.

Adult unknown. Larvae solitary, not forming communal mines as in *A. voraginella*; different timing.

##### Host plants.

Vitaceae: *Vitis arizonica*.

##### Leafmine.

(Figs 84–87) A more or less triangular blotch with scattered black frass, concentrated toward the beginning; no linear part.

##### Larva.

Not examined.

##### Life history.

Larvae collected in November.

##### Distribution.

USA: Arizona.

##### Barcode.

A barcode of 407 bp groups in our tree (Fig. 44) with *A.* “Vitis1_USA”.

##### Remarks.

It is yet not certain whether this species belongs really to *Aspilanta*, as the (incomplete) barcode seems to group more with *Holocacista* species.

##### Material: Larvae and leafmines examined.

USA – **Arizona** • 1 larva (barcoded), vacated mines; Cochise Co., Coronado National Forest, near Chiricahua National Monument; 31.978922N, 109.357056W; alt. 1675 m; 11 Nov. 2012; C.S. Eiseman leg.; *Vitis arizonica*; EventId: CSE–CH1/ CSE–L141; RMNH.INS.29643.P. • leafmines; Santa Cruz Co., Madera Canyon; 31.716527N, 110.87731W; alt. 1589 m; 06 Nov. 2012; C.S. Eiseman leg.; *Vitis arizonica*; EventId: CSE–L140; RMNH.

**Figures 97–106.**
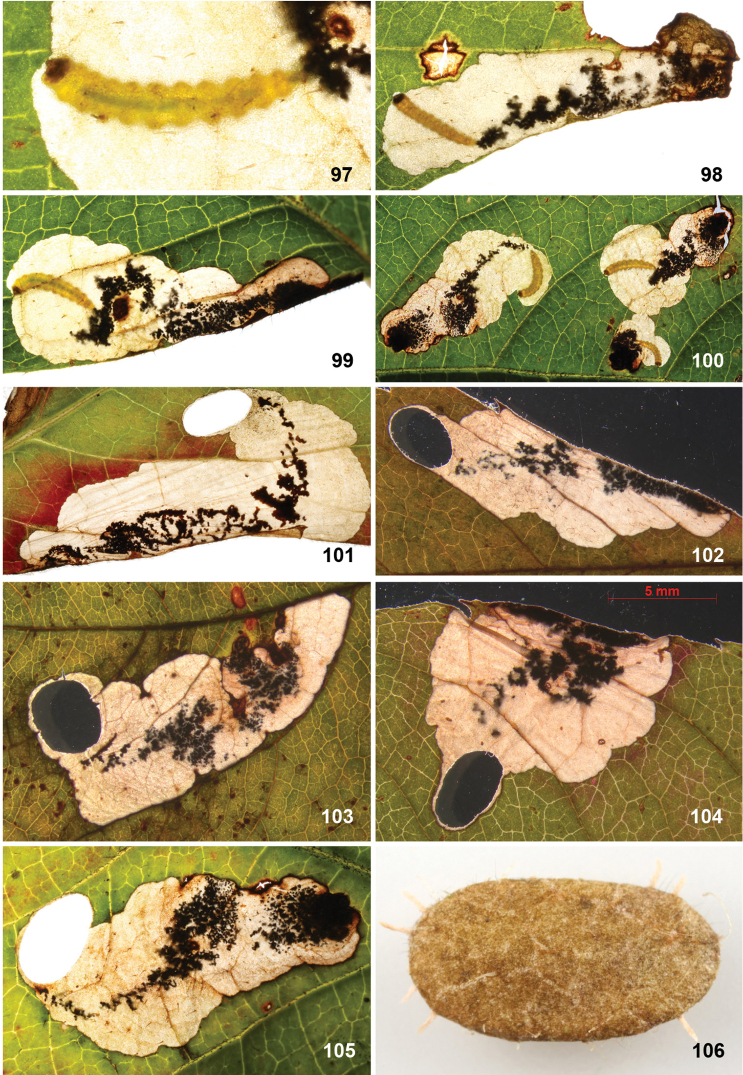
*Aspilanta viticordifoliella*, leafmines, larvae and shields on *Parthenocissus quinquefolia* and *P. vitacea* (104). **97, 99, 100, 105, 106** MA, Northampton, 13.ix.2013, CSE, 105 shows the left mine of 100, 106 showing flat shield to distinguish it from *Antispila* species **98** VT, Williston, 28.viii.2016, CSE **101** MA, Great Barrington, 16.ix.2017, CSE **102, 103** VT, Button Bay SP, 16.ix.2011, EvN2011254–2 **104** QC, Aylmer, 23.viii.2015, EvN2015160.

**Figure 107.**
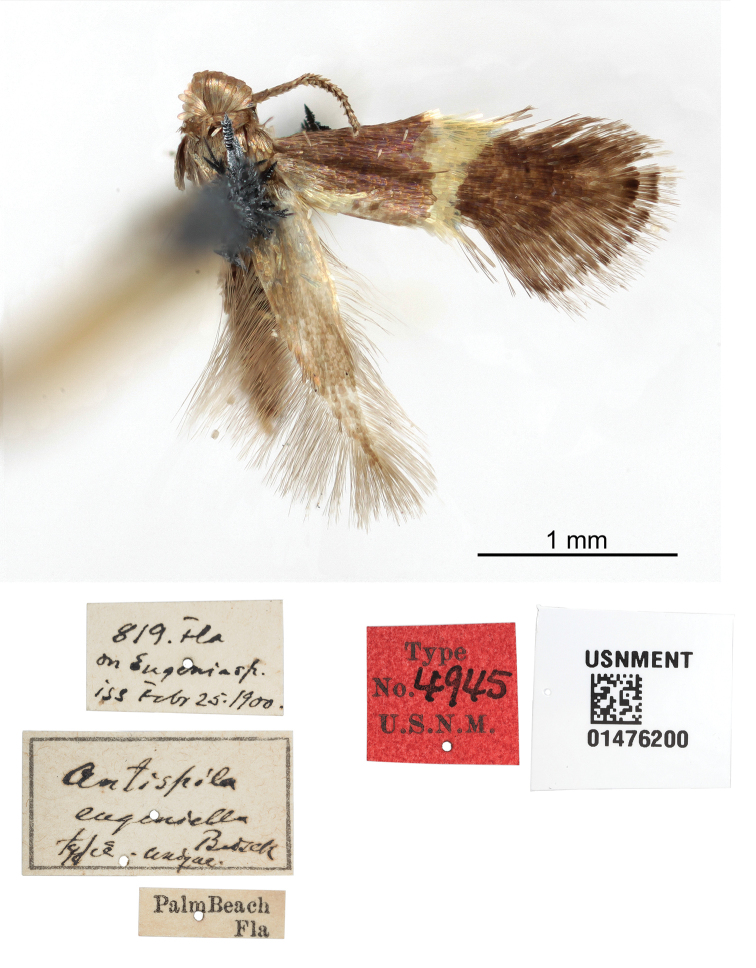
*Heliozela eugeniella*, Holotype with labels. Photograph USNM.

### Discussion

The phylogenetic basis for the erection of the genus *Aspilanta* is the transcriptomic study, which provides a high support for its monophyly (Milla et al. 2019). Unfortunately, we were unable to find more than one possible morphological synapomorphy, the presence of an apical spot in the forewing. Possibly, a more detailed study of immature stages would be able to find more apomorphies. For solving the polyphyly of the genus *Antispila* in the old sense we considered also two alternative options:

1) The inclusion of the whole *Holocacista* group in an enlarged genus *Antispila*. Although this would have solved the problem of polyphyly, it would create a large genus with huge differences in many characters, both encompassing species with reduced venation and the more complete venation, and make recognition of species belonging to this genus almost impossible.

2) Combining all genera of the *Holocacista* group into one genus. Such a genus would be monophyletic and be based on a whole suite of characters, such as the reduced venation. Such a genus should also encompass the undescribed group I (Milla et al. 2017) and the genus14, also known by the manuscript name “*Neospila*” (Milla et al. 2019). The genus should then be named *Coptodisca*, as this is the oldest name. As this name has been in use for more than a century for a rather specialised group of North American leafminers, with a characteristic wing pattern, it would be unfortunate to widen this and include grapevine pests in other parts of the world. Despite the suite of characters, recognition of adults would remain problematic.

The choice of generic borders is in principle not ruled by any other criterion than monophyly, and thus partly subjective. In all three options a number of changes in names are to be expected, but in the chosen option this is limited to just six species, only one of which is an economically important insect. In alternative 1) the number of new combinations would be highest, 26 in total (all *Coptodisca*, *Holocacista* and some others, including five or more economically important species), in 2) still 15 are needed (*Holocacista*, the current *Aspilanta* species, *Antispilina*, *Ischnocanaba*, including at least three economically important species). So in conclusion, the choice for a new genus *Aspilanta* was obvious in terms of stability and recognisability of the groups. Also biogeographically this choice has advantages, as we have now two old world genera (*Holocacista*, *Antispilina*) and two new world genera (*Aspilanta*, *Coptodisca*), whereas *Antispila* remains almost global (unknown from Australia, and so far from the Neotropics). Further study of the Neotropical fauna is important to see whether the monophyly of *Aspilanta* will stand, especially in the light of the position of the Patagonian group of species cited as “*Neospila*”. If they belong to *Aspilanta*, which seems likely morphologically, the paraphyly of the combination Genus14 + *Antispila* group II as noted by Milla et al. (2019) could be problematic. However, it should be noted that the position of Genus14 was poorly supported in that study, and in fact the alternative of a monophyletic Genus14+ *Antispila* group II was found in one of the analyses (Milla et al. 2019: Fig. S5). An analysis comprising several Neotropical species would be necessary for elucidating this phylogeny.

We choose to describe this genus without a full revision of its species. For several of the species we lack sufficient material, and additional fieldwork is much needed, not only for this genus, but for all North American Heliozelidae. Additionally, in *Antispila*, *Coptodisca*, and *Heliozela* we already know of several unnamed species, partly only from Malaise trapped specimens for barcoding campaigns, but also online photographs suggest there are more species. Inclusion of the rich Mexican fauna in such revisions would also be desirable. Further it would be important to settle the status of some old names by dissecting old types while extracting DNA, and for some species to designate neotypes.

## Appendix

By removing six species from *Antispila* to *Aspilanta*, the remaining five named North American species feeding on Cornaceae, Nyssaceae, and Vitaceae belong in the monophyletic genus *Antispila*, as shown by a combination of the molecular studies and their morphology (Milla et al. 2017, 2019). However, one puzzling North American species described in *Antispila* remains: the Myrtaceae-feeding *A. eugeniella* Busck, 1900 from Florida. We here tentatively recombine it with *Heliozela*.

### Heliozela eugeniella

Taxon classificationAnimaliaLepidopteraHeliozelidae

(Busck, 1900) comb. n.

D0C371ED-D934-5FDF-9DDF-517F7A08A891

Fig. 107

Antispila eugeniella Busck, 1900: 236. Holotype adult, USA, Florida, Palm Beach, leafmine on Eugenia sp., bred 25 Feb. 1900, “819”, [leg. Dyar], Type No. 4945, USNMENT 01476200 (USNM).
Antispila eugeniella ; Dyar 1901: 478; Dyar et al. 1903: 539; McDunnough 1939: 91; Kimball 1965: 292; Davis 1983b: 4; Heppner 2003 [2007]: 233; Eiseman 2019: 1153.

#### 

This species is rare; since the unique holotype was reared, we are only aware of a few specimens reared by David L. Wagner (pers. comm.); all subsequent literature records to Dyar (1901) are simple checklist entries. According to Busck (1900) the host plant was *Eugenia* spec., which was according to Dyar (1901), who collected the mine, probably *E. procera* (identified by F. Kinzel), a misapplied name for the Red stopper, *Eugenia rhombea* Krug & Urb. ex Urb. However, we doubt that this identification is correct, as *E. rhombea* is a very rare tree only of the Florida Keys, and not currently occurring in Palm Beach, where only *E. axillaris* (Sw.) Willd. and *E. uniflora* L. have been recorded (Wunderlin and Hansen 2003; Wunderlin et al. 2020). However, we cannot exclude that it did occur there in 1900, as most of the original habitat is now probably lost to development.

We illustrate the holotype here (Fig. 107): a moth with a small white dorsal spot at 1/3 and a fascia at 2/3, which is narrowest at dorsum and widens towards costa.

The colour pattern of *Antispila* and *Aspilanta* is almost always with a fascia at one third and two opposite spots at two-thirds. The pattern of *H. eugeniella* is different, with a postmedial fascia, and resembles more some species of the Old World genus *Holocacista* (van Nieukerken and Geertsema 2015). Most species of *Heliozela* have a basic pattern of two dorsal spots, but there are some species where one or both of these extend towards the costa, e.g., *H. anna* (Fletcher, 1920) from India with two fasciae and *H. argyrozona* (Meyrick, 1918) from South Africa, with a pattern very much resembling that of *H. eugeniella* (van Nieukerken and Geertsema 2015). Both were reared from Myrtaceae, the latter from *Syzygium cordatum* Hochts. ex Krauss, and *H. anna* from *Eugenia jambolana* Lam. (Fletcher 1920), the correct name of which is now *Syzygium cumini* (L.) Skeels (Malabar plum, Jambolan). Also in Australia and Southeast Asia Myrtaceae is a major host family for *Heliozela* (Milla et al. 2017, 2019). These facts suggest that *eugeniella* is better placed in *Heliozela* than in *Antispila*, although still further morphological and molecular confirmation is needed.

## Supplementary Material

XML Treatment for Aspilanta

XML Treatment for Aspilanta oinophylla

XML Treatment for Aspilanta hydrangaeella

XML Treatment for Aspilanta ampelopsifoliella

XML Treatment for Aspilanta voraginella

XML Treatment for Aspilanta argentifera

XML Treatment for Aspilanta viticordifoliella

XML Treatment for Aspilanta

XML Treatment for Aspilanta

XML Treatment for Heliozela eugeniella
